# Spinal **α**2**δ**-1 induces GluA3 degradation to regulate assembly of calcium-permeable AMPA receptors and pain hypersensitivity

**DOI:** 10.1172/JCI193349

**Published:** 2025-10-23

**Authors:** Meng-Hua Zhou, Shao-Rui Chen, Daozhong Jin, Yuying Huang, Hong Chen, Guanxing Chen, Jiusheng Yan, Hui-Lin Pan

**Affiliations:** Center for Neuroscience and Pain Research, Department of Anesthesiology and Perioperative Medicine, The University of Texas MD Anderson Cancer Center, Houston, Texas, USA.

**Keywords:** Aging, Neuroscience, Pain

## Abstract

The increased prevalence of GluA2-lacking, Ca^2+^-permeable AMPA receptors (CP-AMPARs) at spinal cord sensory synapses amplifies nociceptive transmission and maintains chronic neuropathic pain. Nerve injury–induced upregulation of α2δ-1 disrupts the assembly of GluA1/GluA2 heteromers, favoring the synaptic incorporation of GluA1 homotetramers in the spinal dorsal horn. Although GluA1-GluA3 subunits are broadly expressed, whether α2δ-1 regulates GluA3-containing AMPARs remains unknown. Here, we unexpectedly found that coexpression with α2δ-1 — but not α2δ-2 or α2δ-3 — diminished GluA3 AMPAR currents and protein levels, an effect blocked by pregabalin, an α2δ-1 C-terminus peptide, or proteasome inhibition. Both nerve injury and α2δ-1 overexpression reduced protein levels of GluA3 and GluA2/GluA3 heteromers in the spinal cord. Furthermore, α2δ-1 coexpression or nerve injury increased GluA3 ubiquitination, with K861 at the C-terminus of GluA3 identified as a key ubiquitination site mediating α2δ-1–induced GluA3 degradation. Additionally, intrathecal delivery of the *Gria3* gene reversed nerve injury–induced nociceptive hypersensitivity and synaptic CP-AMPARs by restoring protein levels of GluA3 and GluA2/GluA3 heteromers in the spinal cord. These findings reveal that α2δ-1 promotes GluA1 homotetramer assembly and synaptic CP-AMPAR expression by driving ubiquitin-proteasome–mediated degradation of GluA3, providing insights into the molecular mechanisms of neuropathic pain and the therapeutic actions of gabapentinoids.

## Abstract 2

Spinal cord α2δ-1 protein promotes synaptic Ca^2+^-permeable AMPA receptors by degrading GluA3 subunit, revealing how gabapentinoids work and offering new insights into neuropathic pain mechanisms.

## Graphical abstract

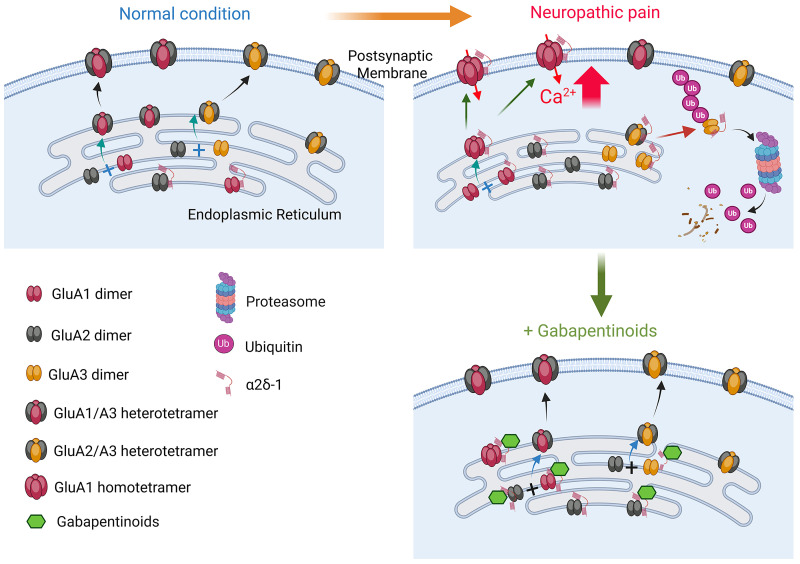

## Introduction

AMPA-type glutamate receptors (AMPARs) are the primary postsynaptic receptors mediating fast excitatory neurotransmission in the central nervous system. Among the four AMPAR subunits (GluA1–GluA4), GluA2 uniquely undergoes Q/R site editing, a modification that critically determines the biophysical properties of AMPARs (1, 2). In the adult brain and spinal cord, most GluA2 proteins contain an arginine (R) residue at position 607, replacing the genomically encoded glutamine (Q607). This positively charged R607 in the pore region prevents Ca^2+^ permeability and results in low single-channel conductance (3). Consequently, GluA2-containing AMPARs are classified as Ca^2+^ impermeable, whereas AMPARs lacking GluA2 are Ca^2+^ permeable (CP-AMPARs) and exhibit distinct inward rectification at positive holding potentials (3–5). Both GluA1 and GluA3 can form homomeric or heteromeric receptors with GluA2. However, unlike GluA1, which readily assembles into homotetramers and traffics to synapses, GluA3 homomers tend to aggregate owing to the presence of 2 key residues — Y454 and R461 — in its ligand-binding domain (6). This aggregation impedes the entry of newly synthesized GluA3 into the secretory pathway and its transport to the cell surface. Notably, GluA3 surface expression is enhanced when coassembled with GluA2 (6). As a result, native AMPARs at glutamatergic synapses predominantly consist of GluA1/GluA2, GluA1/GluA2/GluA3, and GluA2/GluA3 heteromers (7–9). Despite being a major component of synaptic AMPARs in the central nervous system, the physiological and pathological roles of GluA3 remain poorly understood.

GluA1 homomeric receptors, characterized by their high single-channel conductance, constitute the majority of CP-AMPARs and rapidly integrate into the postsynaptic membrane (10). This rapid insertion is crucial for learning and memory as well as pathological processes, such as chronic neuropathic pain (11–13), drug addiction (14), and neurogenic hypertension (15–17). Neuropathic pain is characterized by nociceptive hypersensitivity driven by amplified excitatory input from primary sensory neurons to spinal dorsal horn neurons (18, 19). The hallmark of synaptic plasticity in neuropathic pain is the hyperactivity of *N*-methyl-d-aspartate receptors (NMDARs) and/or CP-AMPARs, which elevate intracellular Ca^2+^ levels at glutamatergic synapses in the spinal dorsal horn (12, 20–22). α2δ-1, encoded by *Cacna2d1* and traditionally known as a voltage-activated Ca^2+^ channel subunit, is upregulated in the dorsal root ganglion and spinal dorsal horn in various neuropathic pain conditions (23–26). Of note, α2δ-1 serves as the primary target of gabapentin and pregabalin (27–29), two widely used drugs for neuropathic pain. Recent studies have identified α2δ-1 as a pivotal regulator of NMDAR trafficking at spinal synapses in neuropathic pain (22, 25, 30). Additionally, α2δ-1 physically interacts with GluA1 and GluA2, disrupting their heteromeric assembly and prompting the synaptic expression of GluA1 homomeric CP-AMPARs at spinal synapses in nerve injury–induced neuropathic pain and calcineurin inhibitor–induced chronic pain (13, 31). However, whether α2δ-1 regulates GluA3-containing AMPARs remains entirely unknown.

In this study, using a heterologous expression system and an animal model of neuropathic pain, we explored how α2δ-1 controls GluA3 proteins in promoting CP-AMPARs. Our findings demonstrate that α2δ-1, via its C-terminal domain, promotes the ubiquitin-proteasome–mediated degradation of GluA3 both in vitro and in vivo. We further identified K861 at the C-terminus of GluA3 as a key site mediating α2δ-1–induced GluA3 ubiquitination. Notably, intrathecal *Gria3* gene delivery reversed nerve injury–induced pain hypersensitivity and synaptic CP-AMPARs by increasing heteromeric GluA2/GluA3 levels in the spinal cord. These findings advance our mechanistic understanding of glutamatergic synaptic plasticity engaged in the persistence of neuropathic pain and provide new insights into the therapeutic mechanisms of gabapentinoids.

## Results

### α2δ-1 coexpression reduces GluA3-containing AMPAR currents in vitro.

**Figure 1:**
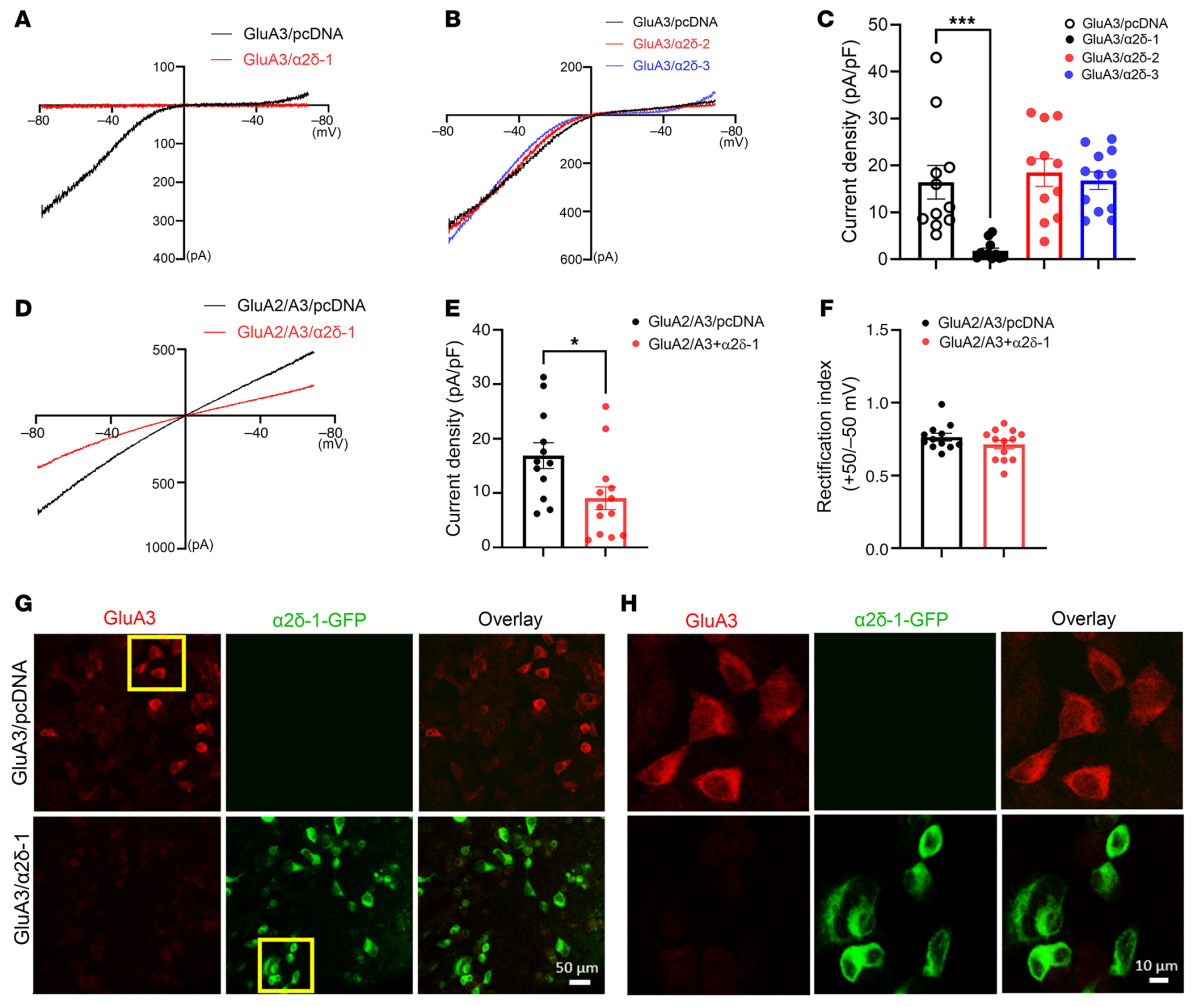
α2δ-1 coexpression diminishes GluA3-containing AMPAR currents and GluA3 protein levels in HEK293 cells. (**A**–**C**) Representative current-voltage (I-V) plots of glutamate-elicited currents (**A** and **B**) and quantification (**C**) show the differential effect of coexpression of α2δ-1, α2δ-2, or α2δ-3 on GluA3 currents in HEK29 cells (*n* = 11 cells for GluA3/empty vector [pcDNA], GluA3/α2δ-1, and GluA3/α2δ-2; *n* = 12 cells for GluA3/α2δ-3). (**D**–**F**) Representative I-V plots of glutamate-elicited currents (**D**) and mean current density (**E**) and rectification index (**F**) in HEK293 cells transfected with GluA2/A3 with either pcDNA or α2δ-1 (*n* = 12 cells for GluA2/GluA3; *n* = 13 cells for GluA2/GluA3/α2δ-1). *P < 0.05, ***P < 0.001; 1-way ANOVA followed by Dunnett’s post hoc test in **C**; 2-tailed Student’s *t* test in **E** and **F**. Data are expressed as means ± SEM. (**G** and **H**) Original confocal immunofluorescence images show the distribution of GluA3 (red) and GFP-tagged α2δ-1 (green) in HEK293 cells transfected with either GluA3/pcDNA or GluA3/α2δ-1-GFP. Areas in yellow boxes in **G** are magnified in **H**. Scale bars: 50 μm (**G**), 10 μm (**H**).

Both GluA1/GluA2 and GluA2/GluA3 heterotetramers are the primary Ca^2+^-impermeable forms of AMPARs in the brain, playing crucial roles in synaptic plasticity and transmission (7, 9, 32). α2δ-1 promotes synaptic incorporation of GluA1 homomeric CP-AMPARs by physically disrupting GluA1/GluA2 heteromeric assembly in the brain and spinal cord (13, 16). To investigate how α2δ-1 controls GluA3-containing AMPARs, we first employed a heterologous expression system to determine whether α2δ-1 coexpression influences GluA3-containing AMPAR currents. In HEK293 cells transfected with GluA3 and empty vectors, the currents elicited by 10 mM glutamate exhibited strong inward rectification, with a current density of 16.45 ± 3.59 pA/pF. Strikingly, coexpression of GluA3 with α2δ-1 at a 1:1 ratio reduced GluA3 currents by 90% [1.80 ± 0.59 pA/pF; *t*_(20)_ = 4.023, *P* = 0.0007; Figure 1, A–C]. However, coexpression of GluA3 with either α2δ-2 or α2δ-3 did not produce a similar inhibitory effect on the GluA3 current density (Figure 1, B and C).

Next, we determined whether α2δ-1 coexpression similarly inhibits AMPAR currents in cells expressing both GluA2 and GluA3 subunits. The AMPAR current density was notably greater in HEK293 cells transfected with GluA2/GluA3 and empty vectors than in those transfected with both GluA2/GluA3 and α2δ-1 (Figure 1, D and E). However, α2δ-1 coexpression did not alter the linear current-voltage relationship or the rectification index of currents mediated by GluA2/GluA3 AMPARs (Figure 1, D and F). These findings demonstrate that α2δ-1 diminishes GluA3-containing AMPAR currents, suggesting a potential regulatory role of α2δ-1 in controlling AMPAR abundance and function.

### α2δ-1, but not α2δ-2 or α2δ-3, diminishes GluA3 protein levels in vitro.

Because α2δ-1 caused a profound reduction in GluA3-containing AMPAR currents, we further examined its effect on GluA3 protein levels using immunofluorescence and immunoblotting analyses. Immunofluorescence labeling showed abundant GluA3 immunoreactivity in HEK293 cells transfected with GluA3 alone. However, in cells cotransfected with GluA3 and α2δ-1 at a 1:1 ratio, GluA3 immunoreactivity was undetectable (Figure 1, G and H).

**Figure 2:**
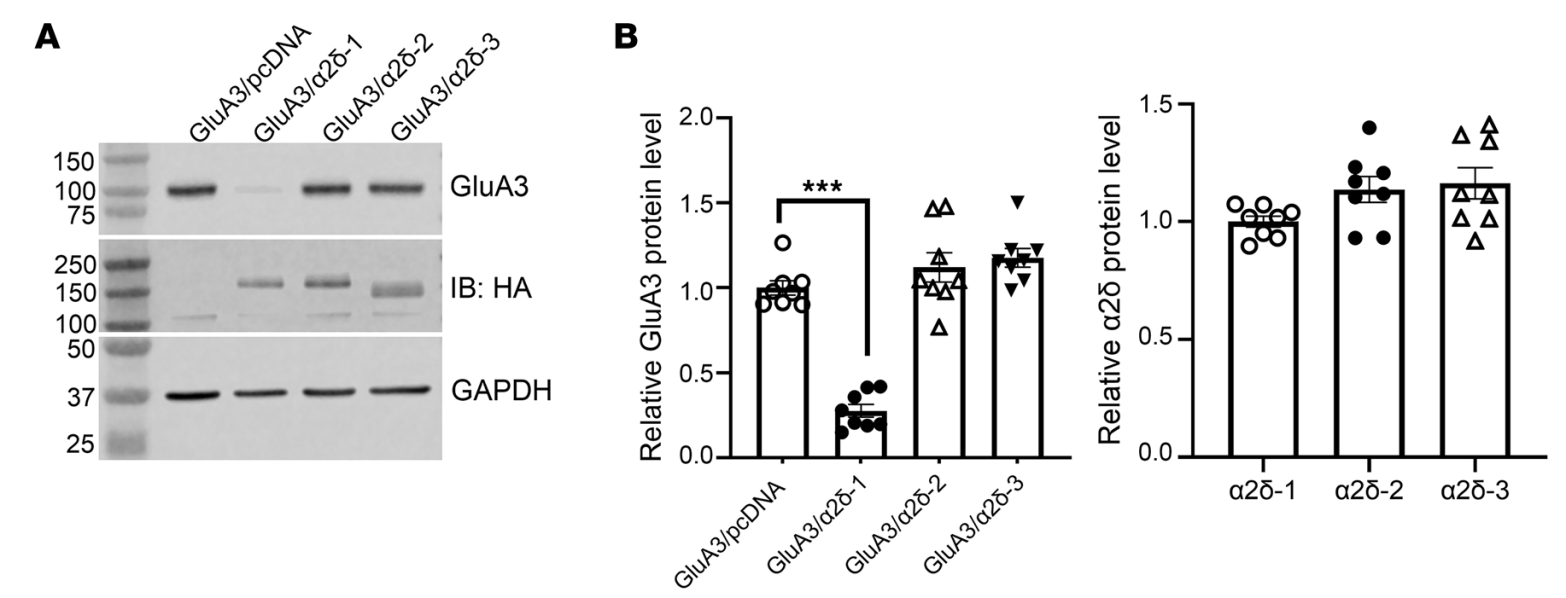
Coexpression of α2δ-1, but not α2δ-2 or α2δ-3, diminishes GluA3 protein levels in vitro. (**A** and **B**) Representative immunoblot images (**A**) and quantification (**B**) show the distinct effect of coexpression of HA-tagged α2δ-1, α2δ-2, or α2δ-3 on GluA3 protein levels in HEK293 cells (*n* = 8 independent experiments per group). GAPDH was used as the internal control for normalizing the protein levels on the same gel. ***P < 0.001; 1-way ANOVA followed by Dunnett’s post hoc test.

**Figure 3:**
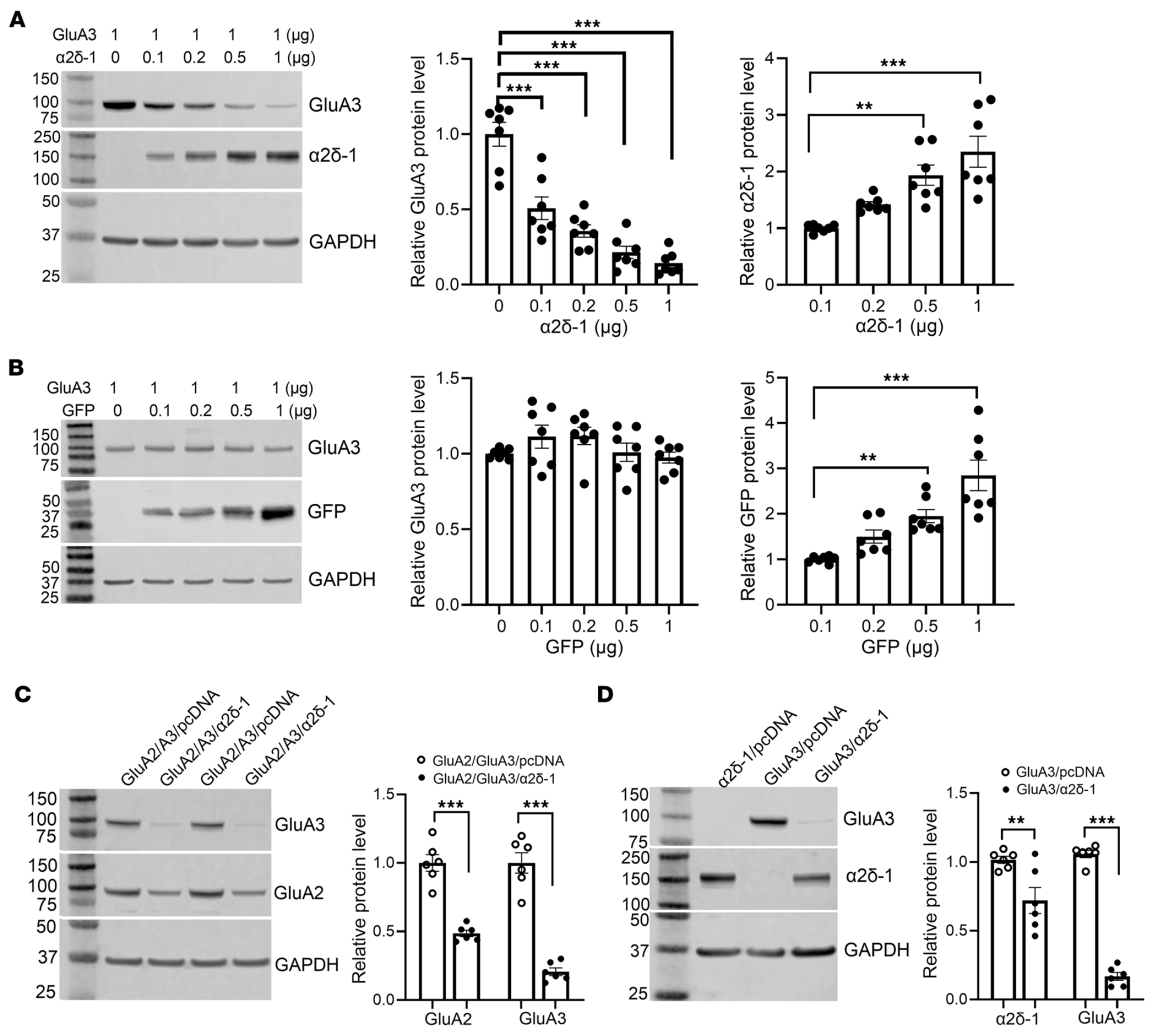
Coexpression of α2δ-1 reduces the levels of GluA3 and GluA2/GluA3 proteins in a concentration-dependent manner in vitro. (**A**) Representative immunoblot images and quantification show the concentration-dependent reduction in GluA3 protein levels induced by α2δ-1 coexpression in HEK293 cells (*n* = 7 independent experiments per group). (**B**) Representative immunoblot images and quantification show the effect of coexpression with GFP on GluA3 protein levels in HEK293 cells (*n* = 7 independent experiments per group). (**C**) Representative immunoblot images and quantification show the protein levels of GluA2 and GluA3 in HEK293 cells expressing GluA2/GluA3 with either empty vectors or α2δ-1 (*n* = 6 independent experiments per group). (**D**) Representative immunoblot images and quantification show the protein levels of GluA3 and α2δ-1 in HEK293 cells expressing GluA3 with either empty vectors or α2δ-1 (*n* = 6 independent experiments per group). GAPDH was used as the internal control for normalizing the protein levels on the same gel. **P < 0.01, ***P < 0.001; 1-way ANOVA followed by Dunnett’s post hoc test in **A** and **B**; 2-tailed Student’s *t* test in **C** and **D**. Data are expressed as means ± SEM.

Consistently, immunoblotting assays confirmed that coexpression of α2δ-1, but not α2δ-2 or α2δ-3, diminished GluA3 protein levels in HEK293 cells (Figure 2, A and B). To determine how α2δ-1 abundance influences GluA3 protein levels, we varied the α2δ-1/GluA3 transfection ratio from 1:1 to 1:10 in HEK293 cells. Immunoblotting analysis revealed that α2δ-1 coexpression reduced GluA3 protein levels in a concentration-dependent manner (Figure 3A). In contrast, cotransfection of GFP and GluA3 at ratios ranging from 1:1 to 1:10 had no effect on GluA3 protein levels in HEK293 cells (Figure 3B). Conversely, GluA3 overexpression concentration-dependently decreased α2δ-1 protein levels (Supplemental Figure 1; supplemental material available online with this article; https://doi.org/10.1172/JCI193349DS1), likely due to the formation of a protein complex between α2δ-1 and GluA3 that undergoes codegradation.

In HEK293 cells cotransfected with GluA2/GluA3 and α2δ-1, α2δ-1 not only diminished GluA3 protein levels but also substantially reduced GluA2 protein levels (Figure 3C). Additionally, both GluA3 and α2δ-1 protein levels were markedly lower in HEK293 cells coexpressing GluA3 and α2δ-1 than in cells expressing either GluA3 or α2δ-1 alone (Figure 3D). These results suggest that α2δ-1 likely promotes the degradation of GluA3 proteins and GluA3-associated protein complexes.

### Nerve injury diminishes GluA3 protein levels in the spinal cord via α2δ-1.

**Figure 4:**
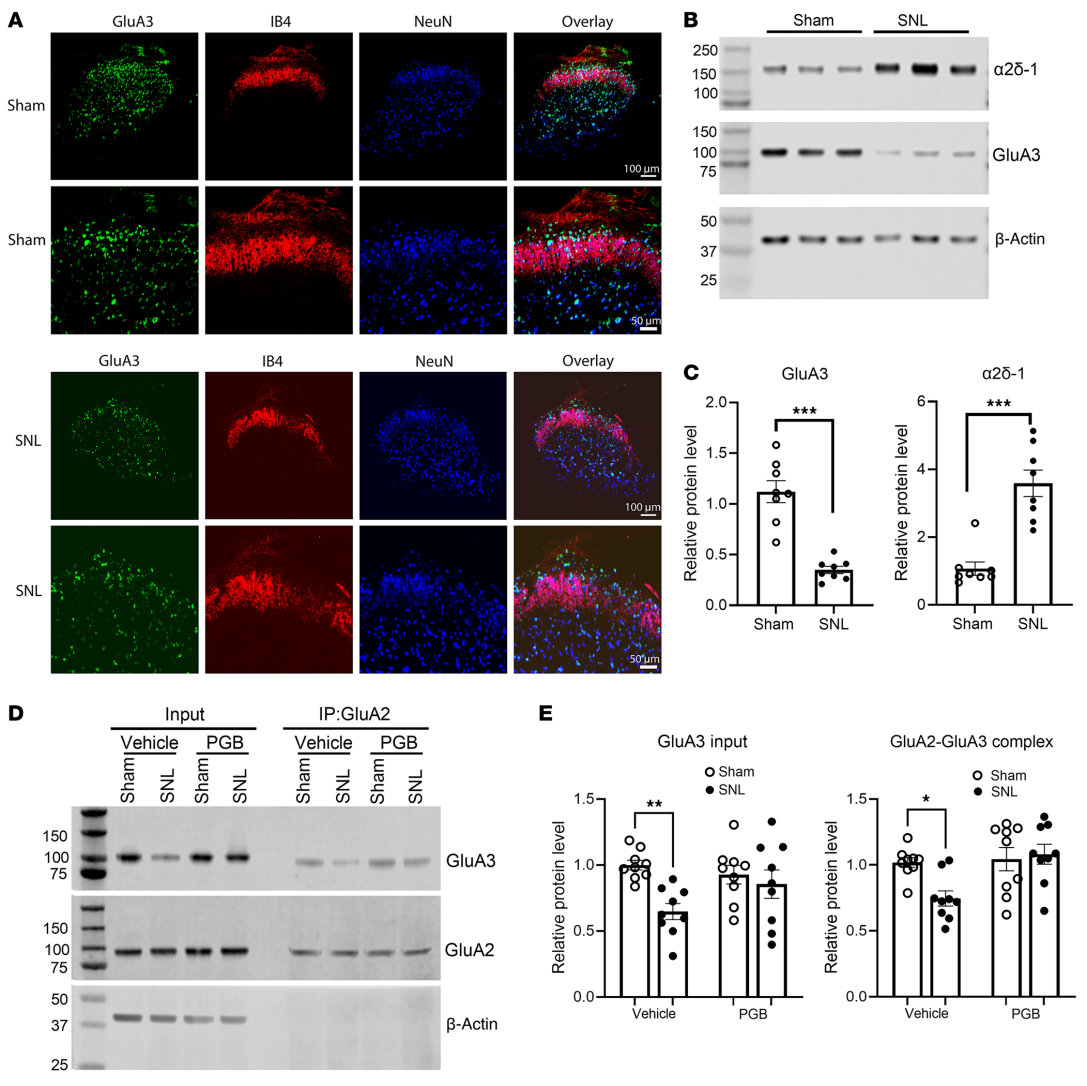
Pregabalin blocks nerve injury–induced reduction in GluA3 and GluA2/GluA3 protein levels in the spinal cord. (**A**) Original confocal images show the distribution of GluA3 (green), IB4 (red), and NeuN (blue) in the spinal dorsal horn of sham control and SNL rats. Scale bars: 100 μm (upper panels), 50 μm (lower panels). (**B** and **C**) Representative immunoblot images (**B**) and quantification (**C**) show the protein levels of α2δ-1 and GluA3 in the dorsal spinal cord of sham control and SNL rats. β-Actin served as the internal control for normalizing the protein levels on the same gel (*n* = 8 mice per group). (**D** and **E**) Representative immunoblot images (**D**) and quantification (**E**) show the protein levels of GluA3 and GluA2/GluA3 complexes in the dorsal spinal cord from sham and SNL rats treated intrathecally with vehicle or 10 μg pregabalin (PGB; *n* = 9 rats per group) 3 weeks after surgery. Protein extracts from rat spinal cord tissues were immunoprecipitated using a rabbit GluA2 antibody or IgG. Immunoblotting was then performed using mouse GluA2, mouse GluA3, and mouse β-actin antibodies. β-Actin served as the internal control for normalizing GluA3 protein levels in the input. The corresponding immunoprecipitated GluA2 protein bands were used for normalizing GluA2/GluA3 protein complex levels. *P < 0.05, **P < 0.01, ***P < 0.001; 2-tailed Student’s *t* test in **C**; 2-way ANOVA followed by Tukey’s post hoc test in **E**. Data are expressed as means ± SEM.

Nerve injury leads to an early and sustained upregulation of α2δ-1 in the dorsal root ganglion and spinal dorsal horn (23, 24, 26). α2δ-1 has a critical role in amplifying nociceptive transmission in neuropathic pain by promoting synaptic expression of NMDARs and GluA1 homomeric CP-AMPARs in the spinal dorsal horn (12, 13, 22). Thus, we determined whether nerve injury affects GluA3 protein levels in the spinal cord. We collected dorsal spinal cord tissues from rats 3 weeks after spinal nerve ligation (SNL) or sham surgery. Immunofluorescence labeling indicated a notable decrease in GluA3 immunoreactivity in the superficial dorsal horn of SNL rats compared with sham controls (Figure 4A). Correspondingly, immunoblotting analysis also demonstrated a large reduction in GluA3 protein levels [*t*_(14)_ = 6.745, *P* < 0.001], accompanied by a concurrent increase in α2δ-1 protein levels [*t*_(14)_ = 5.763, *P* < 0.0001], in the dorsal spinal cord of SNL rats (Figure 4, B and C).

α2δ-1 is the primary target of gabapentinoids (28, 29), such as gabapentin and pregabalin, which are commonly used to treat patients with neuropathic pain and epilepsy. Inhibiting α2δ-1 with gabapentinoids fully restored GluA1/GluA2 heteromeric assembly impaired by α2δ-1 coexpression or nerve injury (13). To determine the role of α2δ-1 in the nerve injury–induced reduction of GluA3 protein levels in the spinal cord, we intrathecally injected 10 μg of pregabalin or vehicle in SNL and sham rats 3 weeks after surgery. This dose of pregabalin effectively attenuates neuropathic pain in rodent models without causing sedation (33, 34). As expected, intrathecal injection of pregabalin readily reversed nociceptive hypersensitivity in SNL rats, reaching a peak effect 60 minutes after injection (Supplemental Figure 2). Spinal cord tissues were collected 60 minutes after injection for immunoblotting analysis. Compared with the vehicle group, pregabalin treatment rescued the SNL-induced reduction in GluA3 protein levels and heteromeric GluA2/GluA3 levels in the spinal cord (Figure 4, D and E).

**Figure 5:**
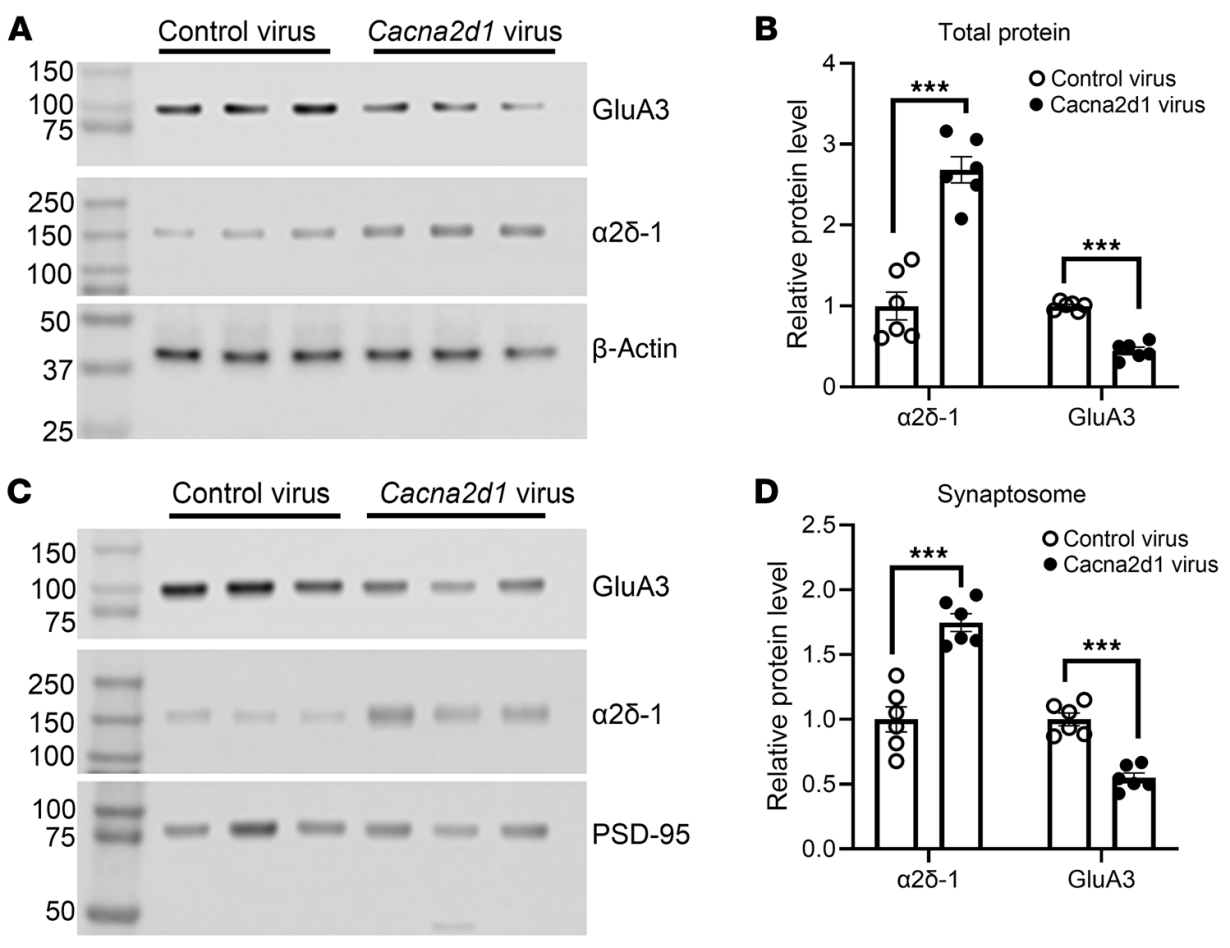
Overexpression of α2δ-1 reduces GluA3 protein levels in the spinal cord. Representative immunoblot images (**A** and **C**) and quantification show the total (**B**) and synaptosome (**D**) protein levels of GluA3 and α2δ-1 in the dorsal spinal cord of naive rats injected intrathecally with control lentiviruses or lentiviruses expressing *Cacna2d1* (*n* = 6 rats per group). β-Actin served as the internal control for normalizing the GluA3 and α2δ-1 protein levels on the same gel. PSD-95, a synaptic protein marker, served as the internal control for normalizing the GluA3 and α2δ-1 protein levels in synaptosome fractions. ***P < 0.001; 2-tailed Student’s *t* test. Data are expressed as means ± SEM.

Furthermore, we determined whether α2δ-1 overexpression affects GluA3 protein levels in vivo by intrathecal injection of *Cacna2d1*-expressing lentiviruses in naive rats (13, 22). As expected, α2δ-1 protein levels in the dorsal spinal cord and spinal synaptosomes were substantially higher in rats receiving *Cacna2d1*-expressing viruses than in those given control viruses. Notably, *Cacna2d1* overexpression markedly reduced GluA3 protein levels in the dorsal spinal cord [*t*_(10)_ = 11.82, *P* < 0.001] and spinal synaptosomal fractions [*t*_(10)_ = 7.337, *P* < 0.0001; Figure 5, A–D]. However, GluA2 protein levels in the dorsal spinal cord were comparable between the 2 groups (Supplemental Figure 3), suggesting that a substantial portion of GluA2 is likely associated with GluA1 in vivo.

**Figure 6:**
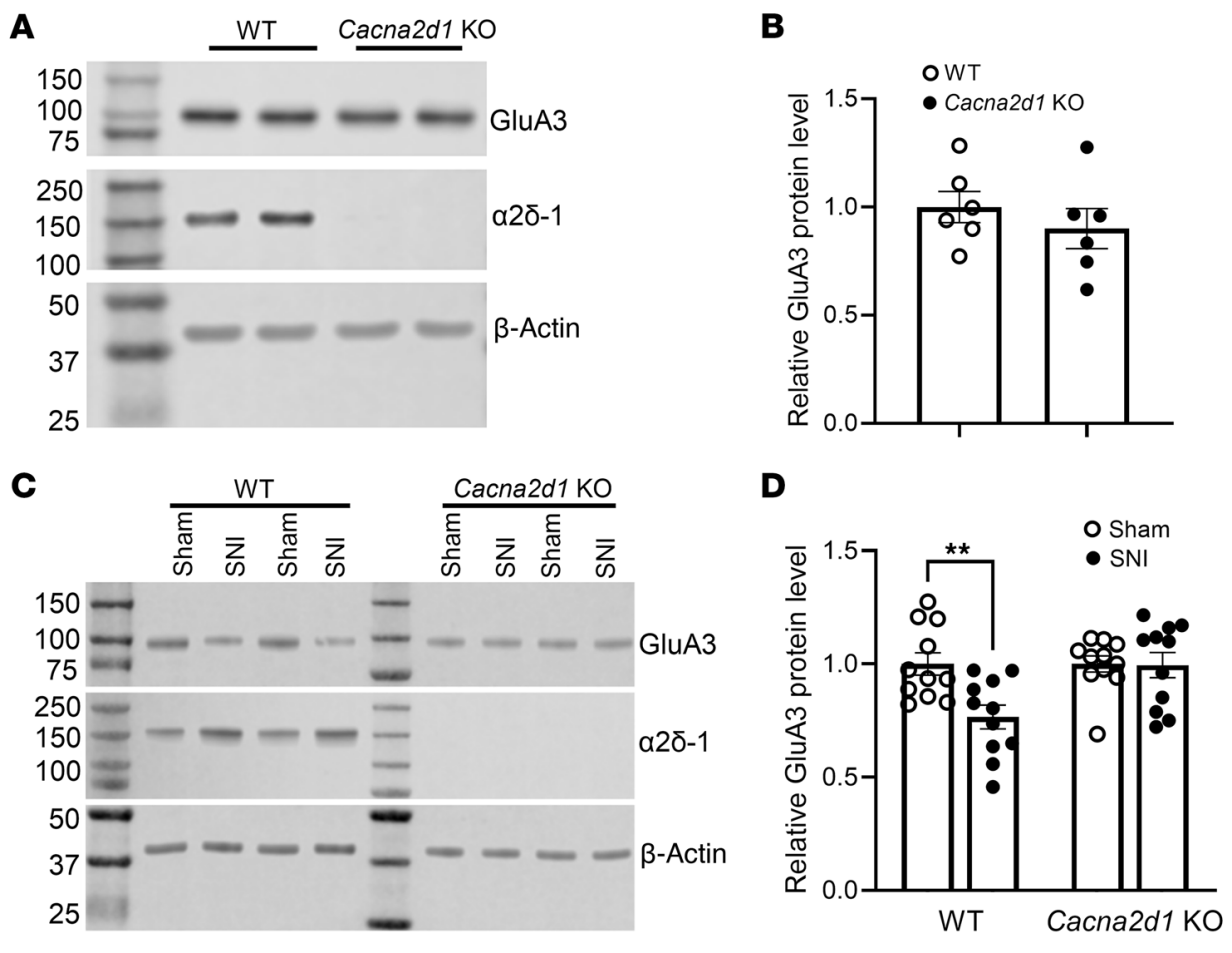
Role of α2δ-1 in nerve injury–induced reduction of GluA3 protein levels in the spinal cord. (**A** and **B**) Representative immunoblot images (**A**) and quantification (**B**) show the basal protein levels of GluA3 in the dorsal spinal cord of WT and *Cana2d1-*KO mice (*n* = 6 mice per group). (**C** and **D**) Representative immunoblot images (**C**) and quantification (**D**) show the protein levels of GluA3 and α2δ-1 in dorsal spinal cord tissues from WT and *Cana2d1-*KO mice subjected to sham or SNI surgery (*n* = 11 mice per group). β-Actin served as the internal control for normalizing the GluA3 and α2δ-1 protein levels on the same gel. **P < 0.01; 2-tailed Student’s *t* test. Data are expressed as means ± SEM.

In addition, we used *Cacna2d1*-KO mice to validate the role of α2δ-1 in nerve injury–induced reduction of spinal cord GluA3 protein levels. Compared with WT controls, *Cacna2d1* KO exhibited a small effect on the development of nociceptive hypersensitivity after nerve injury (Supplemental Figure 4). Immunoblotting analysis showed no difference in baseline GluA3 protein levels in the dorsal spinal cord between *Cacna2d1*-KO and WT mice (Figure 6, A and B). However, spared nerve injury (SNI) markedly reduced GluA3 protein levels in the spinal cord of WT mice, but not in *Cacna2d1*-KO mice (Figure 6, C and D). These findings provide compelling in vivo evidence that α2δ-1 is essential for nerve injury–induced GluA3 protein degradation in the spinal cord.

### α2δ-1 induces GluA3 degradation via its C-terminus.

**Figure 7:**
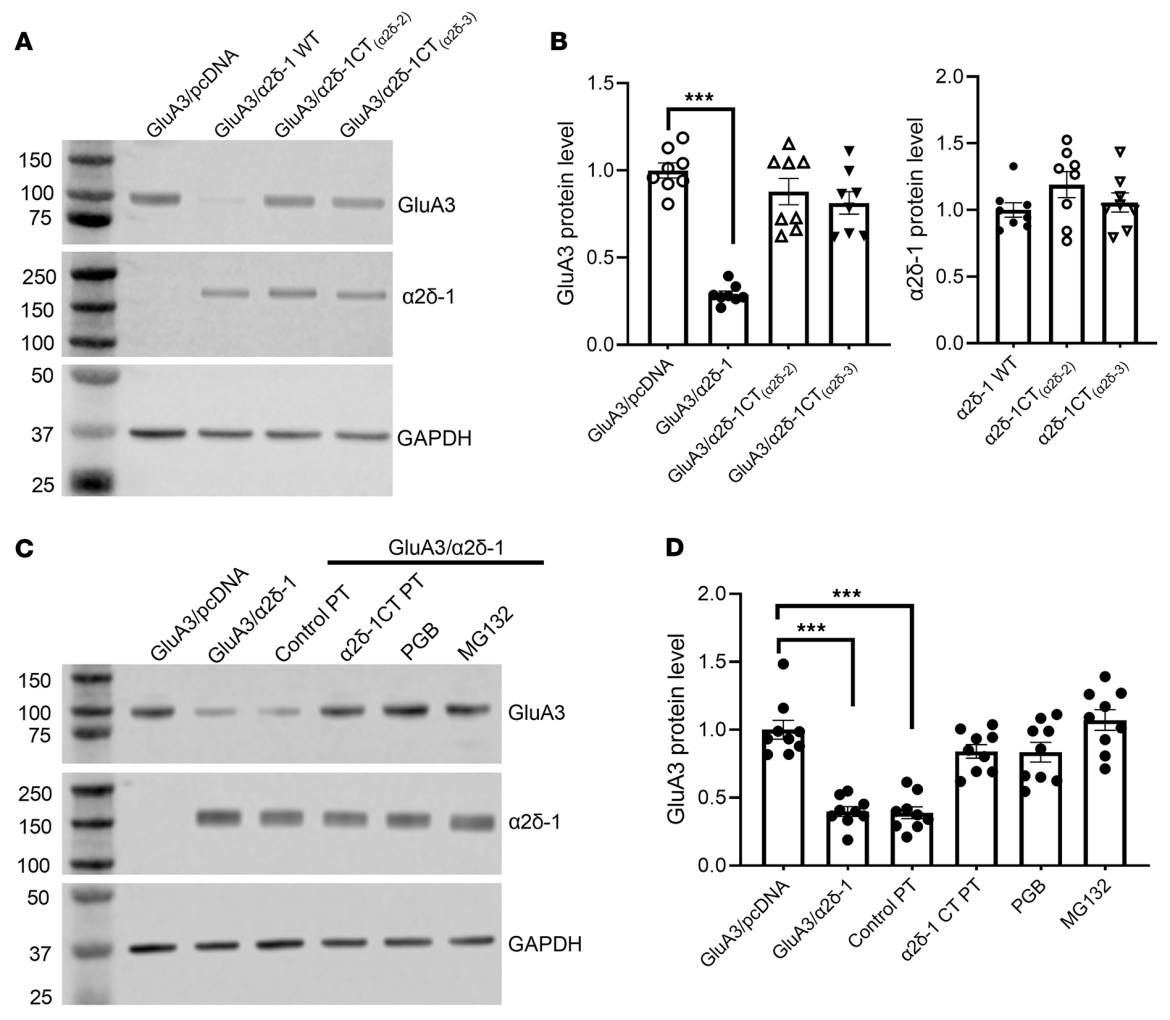
α2δ-1 induces GluA3 degradation through its C-terminus. (**A** and **B**) Representative immunoblot images (**A**) and quantification (**B**) show GluA3 and α2δ-1 protein levels in HEK293 cells coexpressing GluA3 with YFP-tagged WT α2δ-1 or chimeric constructs [α2δ-1CT_(α2δ-2)_ and α2δ-1CT_(α2δ-3)_] (*n* = 8 independent experiments per group). (**C** and **D**) Representative immunoblot images (**C**) and quantification (**D**) show the effects of treatment with control peptide (1 μM), α2δ-1CT peptide (1 μM), pregabalin (PGB; 20 μM), and MG132 (10 μM) on the GluA3 protein levels in HEK293 cells coexpressing α2δ-1 and GluA3 (*n* = 9 independent experiments per group). PT, peptide. GAPDH was used as an internal control for normalizing the GluA3 protein levels on the same gel. ***P < 0.001; 1-way ANOVA followed by Dunnett’s post hoc test. Data are expressed as means ± SEM.

We next attempted to identify the molecular determinant responsible for α2δ-1–induced GluA3 protein degradation. α2δ-1 physically interacts with GluA1 and GluA2 via its C-terminal domain (13). Because α2δ-2 and α2δ-3 did not affect GluA3 protein levels, we used chimeric constructs — α2δ-1CT_(α2δ-2)_ and α2δ-1CT_(α2δ-3)_ — in which the C-terminal domain of α2δ-1 was replaced with the corresponding domain from α2δ-2 or α2δ-3 (22). HEK293 cells were transfected with GluA3 and YFP-tagged WT α2δ-1, α2δ-1CT_(α2δ-2)_, or α2δ-1CT_(α2δ-3)_. Immunoblotting assays revealed that coexpression of WT α2δ-1, but not the chimeric α2δ-1CT_(α2δ-2)_ or α2δ-1CT_(α2δ-3)_, diminished GluA3 protein levels (Figure 7, A and B).

To further investigate the molecular mechanism by which α2δ-1 induces GluA3 degradation, we determined the effects of pregabalin and a Tat-fused α2δ-1 peptide that mimics the C-terminal domain of α2δ-1 (α2δ-1CT peptide), which competitively blocks the interaction of full-length α2δ-1 proteins with NMDARs, GluA1, and GluA2 (13, 17, 22, 35). We have demonstrated that intrathecal injection of α2δ-1CT peptide attenuates pain hypersensitivity caused by nerve injury (13, 22, 30). Overnight treatment (14 h) with 20 μM pregabalin or 1 μM α2δ-1CT peptide, but not 1 μM control peptides, largely reversed the α2δ-1–induced reduction in GluA3 protein levels in HEK293 cells (Figure 7, C and D). These data provide strong evidence that the C-terminus of α2δ-1 is essential for α2δ-1–induced proteasomal degradation of GluA3 proteins.

### Proteasome inhibition reverses GluA3 degradation caused by α2δ-1 coexpression or nerve injury.

To determine whether proteasomes are involved in α2δ-1–induced GluA3 degradation, we treated transfected HEK293 cells with 10 μM MG132, a potent and cell-permeable proteasome inhibitor (36, 37). MG132 treatment for 14 h effectively prevented the reduction in GluA3 protein levels induced by α2δ-1 coexpression (Figure 7, C and D).

**Figure 8:**
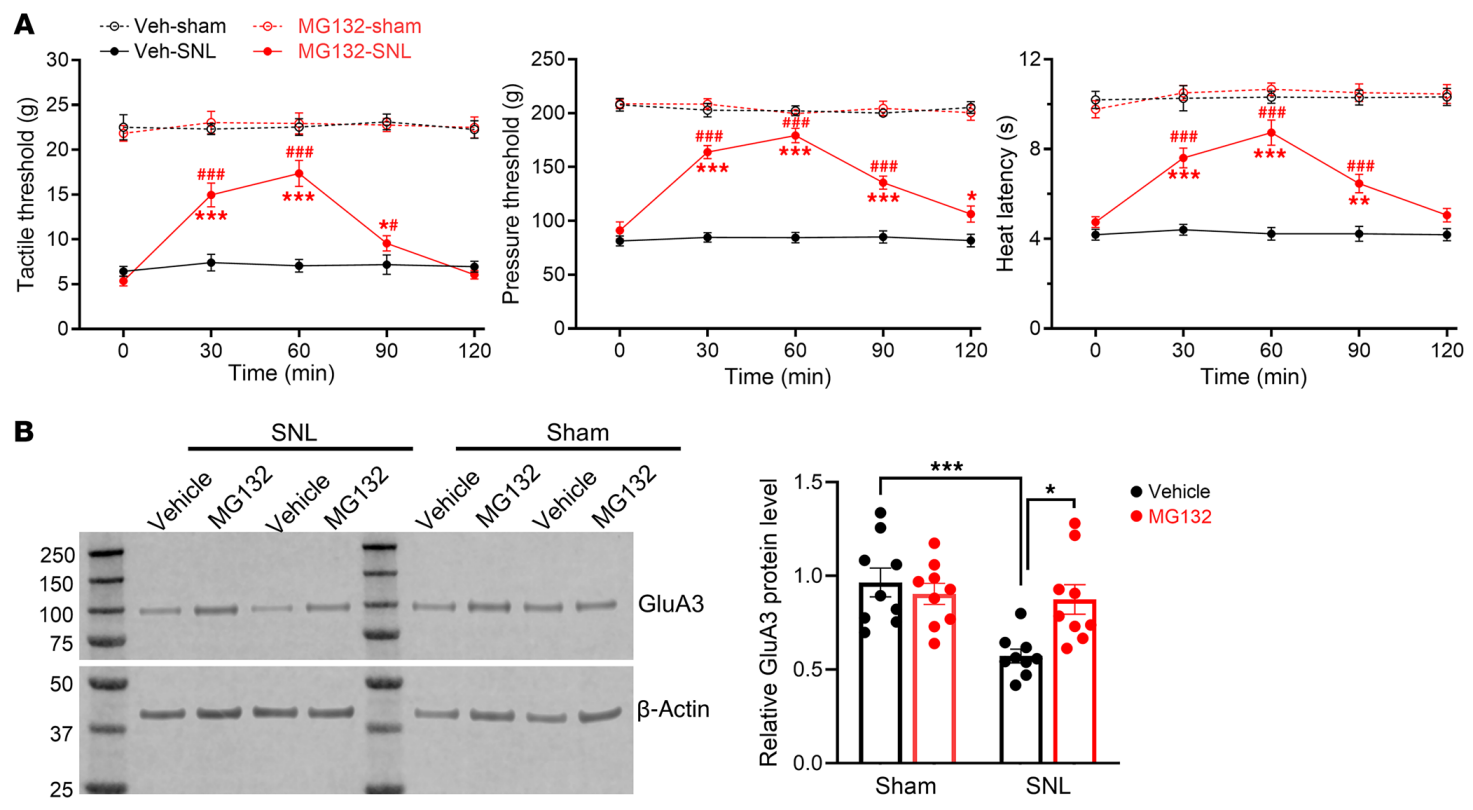
Proteasome inhibition reverses nerve injury–induced reductions in nociceptive thresholds and GluA3 protein levels in the spinal cord. (**A**) Time-course effects of intrathecal injection of 20 μg MG132 or vehicle (Veh) on hindpaw nociceptive thresholds in sham and SNL rats 3 weeks after surgery (*n* = 9 rats per group). *P < 0.05, **P < 0.01, ***P < 0.001 versus baseline (time 0); ^#^*P* < 0.05, ^###^*P* < 0.001 versus Veh-SNL group at the same time point; 2-way ANOVA followed by Tukey’s post hoc test. (**B**) Representative immunoblot images and quantification show the effect of MG132 treatment on GluA3 protein levels in dorsal spinal cord tissues from SNL and sham rats (*n* = 9 rats per group). *P < 0.05, ***P < 0.001; 1-way ANOVA followed by Tukey’s post hoc test. Data are expressed as means ± SEM.

We then determined whether proteasomal inhibition could also rescue nerve injury–induced GluA3 reduction in the spinal cord. Inhibiting the ubiquitin-proteasome system in the spinal cord reduces nerve injury–induced pain hypersensitivity (38, 39). Intrathecal injection of 20 μg MG132 markedly attenuated nociceptive hypersensitivity in SNL rats but had no such effect in sham controls (Figure 8A). Spinal cord tissues were collected from SNL and sham rats 60 minutes after MG132 or vehicle injection. Immunoblotting analysis showed that treatment with MG132, but not vehicle, restored the GluA3 protein levels reduced by SNL (Figure 8B). These results support the critical role of the proteasome system in GluA3 reduction induced by either α2δ-1 coexpression or nerve injury.

### α2δ-1 coexpression or nerve injury increases GluA3 protein ubiquitination in vitro and in vivo.

**Figure 9:**
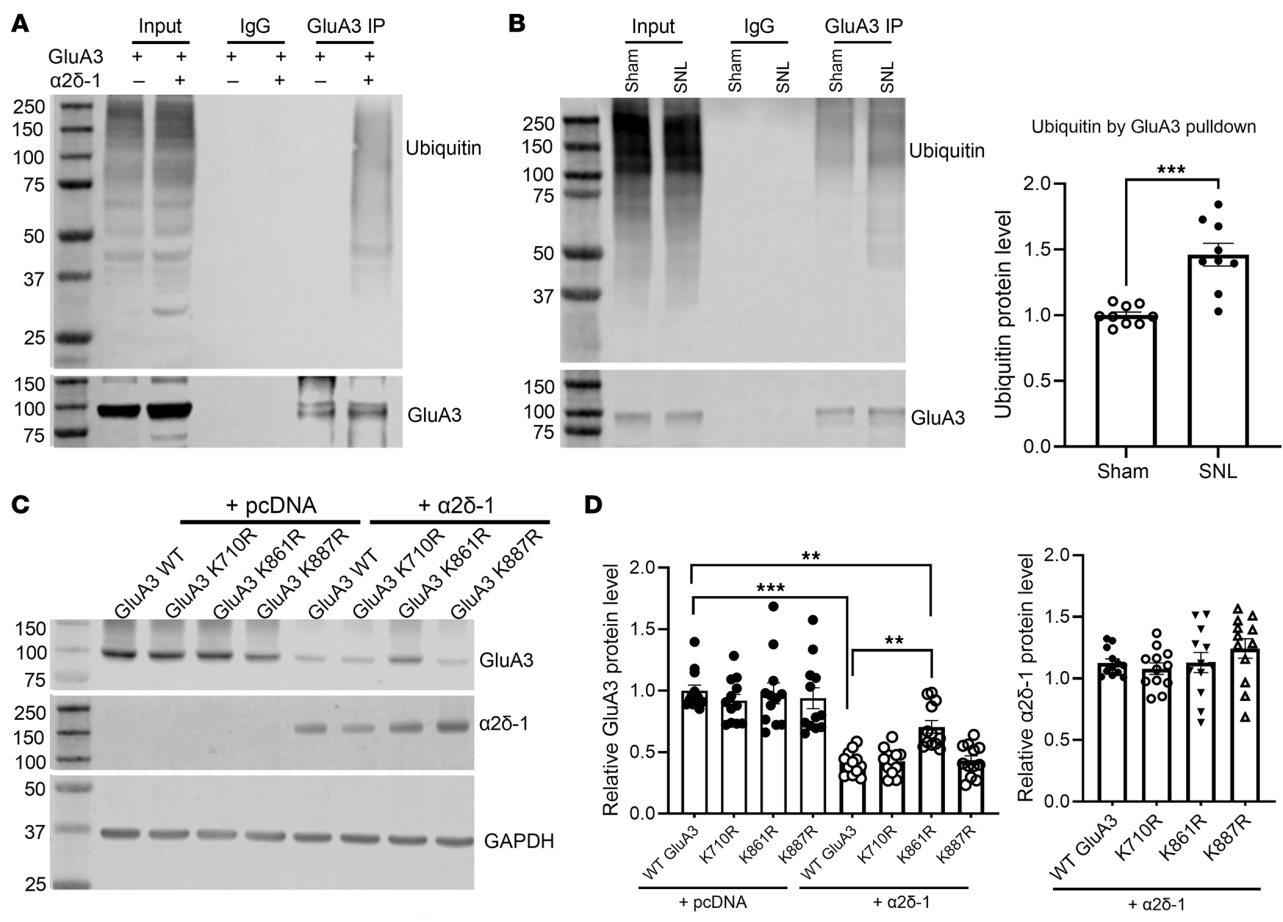
α2δ-1 coexpression or nerve injury enhances GluA3 protein ubiquitination in vitro and in vivo. (**A**) Representative immunoblot images show the ubiquitin protein levels in GluA3 precipitates from HEK293 cells expressing GluA3 with either pcDNA or α2δ-1 (similar data were obtained from 4 independent experiments). (**B**) Representative immunoblot images and quantification show the ubiquitin protein levels in GluA3 precipitates from the dorsal spinal cord of sham control and SNL rats (*n* = 9 rats per group). Protein extracts from HEK293 cells or spinal cord tissues were immunoprecipitated using a rabbit GluA3 antibody or IgG. Immunoblotting was then conducted using mouse ubiquitin or mouse GluA3 antibodies. The corresponding GluA3 protein bands were used as the internal control on the same gel. (**C** and **D**) Representative immunoblot images (**C**) and quantification (**D**) show GluA3 protein levels in HEK293 cells expressing WT GluA3 or GluA3 mutants (K710R, K861R, and K887R) with and without α2δ-1 (*n* = 12 independent experiments per group). GAPDH was used as the internal control for normalizing GluA3 and α2δ-1 protein levels on the same gel. **P < 0.01, ***P < 0.001; 2-tailed Student’s *t* test in **B**; 1-way ANOVA followed by Tukey’s post hoc test in **D**. Data are expressed as means ± SEM.

To determine whether α2δ-1 coexpression enhances GluA3 ubiquitination, we subsequently examined the levels of ubiquitinated GluA3 proteins. Ubiquitination is a posttranslational modification in which a 76–amino acid ubiquitin molecule is attached to lysine residues of a substrate protein. To mitigate excessive GluA3 degradation caused by α2δ-1 coexpression in HEK293 cells, we reduced the α2δ-1/GluA3 transfection ratio from 1:1 to 1:5, based on our findings above (Figures 2 and 3). We conducted coimmunoprecipitation using an anti-GluA3 antibody for the pulldown and then blotted the GluA3 precipitates with an antiubiquitin antibody. This analysis revealed that the ubiquitin protein was not detected in the GluA3 precipitates from HEK293 cells transfected with GluA3 alone. In contrast, abundant ubiquitin protein levels were present in the GluA3 precipitates from cells cotransfected with both GluA3 and α2δ-1 (Figure 9A).

Next, we determined whether nerve injury potentiates GluA3 ubiquitination in the spinal cord. Coimmunoprecipitation analysis showed that the ubiquitin protein levels in GluA3 precipitates from the dorsal spinal cord were much higher in SNL rats than in sham control rats [*t*_(16)_ = 5.074, *P* < 0.001; Figure 9B]. These findings indicate that α2δ-1 coexpression or nerve injury strongly promotes GluA3 ubiquitination in vitro and in vivo.

### The C-terminal K861 of GluA3 is involved in α2δ-1–mediated GluA3 ubiquitination.

Next, we attempted to identify the lysine residues of GluA3 involved in ubiquitination caused by α2δ-1 coexpression. The primary method for ubiquitinome analyses relies on immunoaffinity purification and mass spectrometry–based (MS-based) detection of diglycine-modified peptides (40). In our experiments, we first used the anti-Flag antibody to purify the samples from HEK293 cells cotransfected with Flag-tagged GluA3 and α2δ-1. We then used MS to identify the putative ubiquitination sites after trypsin digestion. We identified 10 putative diglycine-modified peptides of GluA3 as candidates of ubiquitination sites (Table 1 and Supplemental Figure 5). Putative ubiquitinated peptides of α2δ-1 were rarely detected, likely due to its much lower expression compared with GluA3 (1:3 ratio in transfected plasmids and 1:3.4 ratio in Mascot scores). Given the predominantly extracellular localization of α2δ-1, further attempts to identify its ubiquitinated peptides were not pursued.

Because GluA1 and GluA2 are not directly degraded by α2δ-1 coexpression, we conducted a sequence alignment of GluA1, GluA2, and GluA3 (Supplemental Figure 6). This analysis revealed a high degree of conservation in most lysine (K) residues within candidate ubiquitination sites across the 3 proteins. Notably, GluA3 uniquely contains K710 (corresponding to R in GluA1 and GluA2) and K861 (corresponding to asparagine [N] in GluA1 and GluA2).

To investigate their role, we generated point mutations at K710 and 2 C-terminal lysine residues (K861 and K887), substituting lysine with R. K887, which is conserved between GluA2 and GluA3, served as a control. Immunoblotting analysis of HEK293 cells coexpressing α2δ-1 with either WT GluA3 or GluA3 mutants revealed that K861R, but not K710R or K887R, partially rescued the α2δ-1–induced reduction in GluA3 levels (Figure 9, C and D). These findings suggest that ubiquitination of the C-terminal K861 residue plays an important role in α2δ-1–mediated GluA3 degradation.

### GluA3 abundance controls nerve injury–induced pain hypersensitivity and synaptic CP-AMPARs in the spinal cord.

Our findings above indicate that nerve injury induces proteasomal degradation of GluA3 proteins in the spinal cord through α2δ-1. To determine the functional significance of the GluA3 abundance in neuropathic pain and spinal cord CP-AMPARs induced by nerve injury, we intrathecally injected *Gria3*-expressing lentiviruses or control lentiviruses in sham and SNL rats 2 weeks after surgery. We did not use the GluA3 K861R mutant for overexpression because it only partially rescued α2δ-1–induced GluA3 degradation. In addition, this mutation may alter GluA3’s interactions or assembly with GluA2 as well as their channel activity.

**Figure 10:**
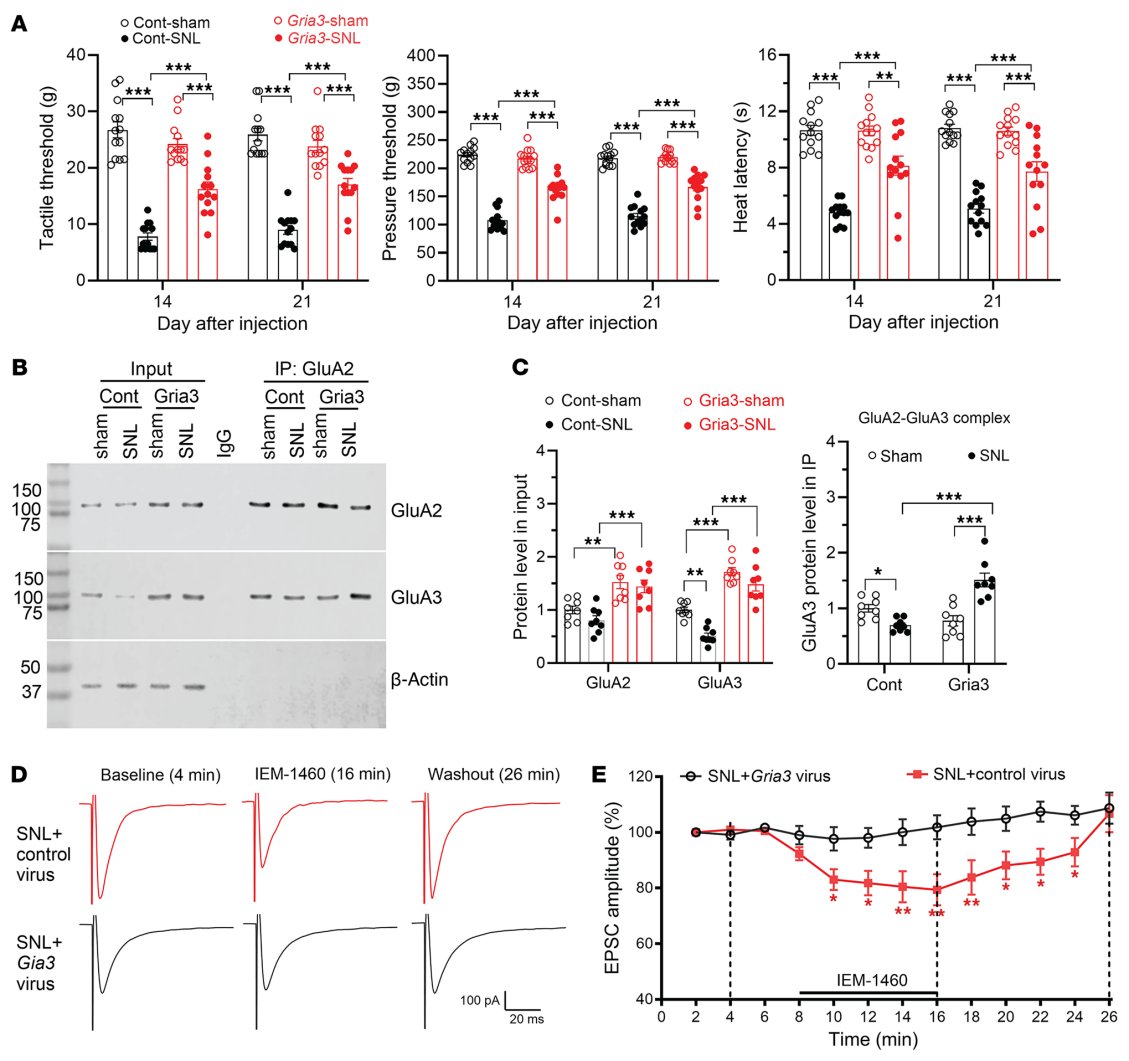
Intrathecal *Gria3* delivery reverses nerve injury–induced pain hypersensitivity and synaptic CP-AMPARs by promoting GluA2/GluA3 formation in the spinal cord. (**A**) Changes in the hindpaw withdrawal thresholds of sham control and SNL rats 2 and 3 weeks after intrathecal injection of control (Cont) lentiviral vectors or lentiviral vectors expressing *Gria3* (*n* = 13 rats per group). **P < 0.01, ***P < 0.001; 2-way ANOVA followed by Tukey’s post hoc test. (**B** and **C**) Representative immunoblot images (**B**) and quantification (**C**) show the protein levels of GluA3 and GluA2/GluA3 complexes in the dorsal spinal cords of sham control and SNL rats treated with intrathecal control lentiviruses or *Gria3*-expressing lentiviruses (*n* = 8 rats per group). Protein extracts from spinal cord tissues were immunoprecipitated (IP) using a GluA2 antibody or IgG. Immunoblotting was then conducted using GluA2, GluA3, and β-actin antibodies. β-Actin protein bands were used as the internal control on the same gel. **P < 0.01, ***P < 0.001; 2-way ANOVA followed by Tukey’s post hoc test. (**D** and **E**) Representative recording traces (**D**) and quantification (**E**) show the differential effect of bath application of IEM-1460 (50 μM) on the amplitude of monosynaptic AMPAR-EPSCs in spinal lamina II neurons from SNL rats treated with intrathecal injection of control lentiviruses or *Gria3*-expressing lentiviruses (*n* = 18 neurons from 4 rats per group). Data were normalized to the baseline value (100%) before IEM-1460 application. *P < 0.05, **P < 0.01 versus control vector group at the same time point; 2-way ANOVA followed by Tukey’s post hoc test. Data are presented as mean ± SEM.

In sham control rats, the hindpaw withdrawal thresholds did not differ significantly between those treated with *Gria3*-expressing viruses and those treated with control viruses. However, compared with SNL rats injected with control viruses, rats receiving *Gria3*-expressing viruses exhibited a substantial attenuation of SNL-induced reductions in mechanical and thermal withdrawal thresholds, without any overt behavioral abnormalities at 2 and 3 weeks after injection (Figure 10A). Immunoblotting analysis confirmed that GluA3 protein levels in the spinal cord of both sham and SNL rats were markedly higher in rats treated with *Gria3*-expressing viruses than in rats treated with control viruses (Figure 10, B and C). Interestingly, treatment with *Gria3*-expressing viruses also increased GluA2 protein levels in the spinal cord of both sham and SNL rats (Figure 10, B and C). Furthermore, coimmunoprecipitation analysis showed that compared with rats treated with control viruses, treatment with *Gria3*-expressing viruses increased the GluA2/GluA3 complex levels in the spinal cord of SNL rats but not sham rats (Figure 10, B and C).

Additionally, we conducted whole-cell recordings of AMPAR-mediated excitatory postsynaptic currents (EPSCs) in spinal cord slices from SNL rats 3 weeks after intrathecal injection of *Gria3*-expressing viruses or control viruses. Monosynaptic EPSCs in lamina II neurons were evoked by dorsal root stimulation (11, 12). After obtaining a stable baseline EPSC, we bath applied 100 μM IEM-1460, a specific open-channel blocker of CP-AMPARs (13, 41). We have shown that IEM-1460 has no significant effect on evoked EPSCs of spinal dorsal horn neurons from sham control rats (13). In SNL rats injected with control viruses, bath application of IEM-1460 for 8 minutes caused a large reduction in the baseline amplitude of EPSCs in lamina II neurons (Figure 10, D and E), indicating the presence of synaptic CP-AMPARs in the spinal cord, as previously reported (12, 13). In contrast, bath application of IEM-1460 had no inhibitory effect on the baseline amplitude of EPSCs in lamina II neurons from SNL rats treated with *Gria3*-expressing viruses (Figure 10, D and E). These results suggest that nerve injury–induced GluA3 protein degradation contributes to the persistence of neuropathic pain and the increased prevalence of synaptic CP-AMPARs in the spinal cord.

## Discussion

Our study provides strong in vitro and in vivo evidence revealing that α2δ-1 promotes the degradation of GluA3 proteins. AMPARs are tetrameric ligand-gated ion channels formed by combinations of GluA1–GluA4 subunits, with the primary AMPAR complexes in the brain being GluA1/GluA2, GluA1/GluA2/GluA3, and GluA2/GluA3 (7, 9, 10). The relative abundance and subunit composition of AMPARs vary across different brain regions (42, 43). GluA1/GluA2 AMPARs are primarily inserted into the postsynaptic membrane in response to synaptic activity, whereas GluA2/GluA3 AMPARs undergo constitutive recycling at synapses (32). Importantly, AMPARs often coassemble with various interacting proteins that control trafficking, localization, kinetics, and pharmacology of the assembled AMPAR complexes (7, 10). α2δ-1 facilitates GluA1 homomeric AMPARs at spinal synapses in neuropathic pain by directly interacting with both GluA1 and GluA2 to disrupt their heteromeric assembly (13). In this study, we unexpectedly found that coexpression of α2δ-1 diminished GluA3 or GluA2/GluA3 currents in a cell line. Furthermore, α2δ-1 coexpression markedly reduced GluA3 protein levels, as well as α2δ-1 and GluA2 proteins complexed with GluA3. HEK293 cells lack functional NMDARs, so these results indicate that α2δ-1 induces GluA3 degradation independently of NMDARs. Consistent with the in vitro findings, α2δ-1 overexpression similarly diminished GluA3 proteins in the spinal cord. We also found that nerve injury led to a profound reduction in GluA3 and GluA2/GluA3 heteromer levels in the spinal cord, an effect that was blocked by α2δ-1 KO. α2δ-1 coexpression or nerve injury does not reduce the protein levels of GluA1 and GluA2 (12, 13), so this α2δ-1–induced AMPAR degradation appears to be GluA3 subtype specific. Interestingly, we found that α2δ-1 coexpression failed to induce GluA3 degradation when the C-terminus of α2δ-1 was replaced with the corresponding domain from α2δ-2 or α2δ-3, highlighting the essential role of the C-terminus of α2δ-1 in α2δ-1–induced GluA3 degradation. These striking findings indicate that α2δ-1 directly interacts with GluA3 through its C-terminus, triggering the proteasomal degradation of α2δ-1–GluA3 and α2δ-1–GluA2/GluA3 protein complexes.

In this study, we demonstrated that α2δ-1 induces GluA3 degradation via the ubiquitin-proteasome pathway. Ubiquitination is a reversible posttranslational modification that involves the covalent attachment of a 76–amino acid ubiquitin to lysine residues of target proteins, regulating various processes, including protein degradation, receptor sorting, endocytosis, and synaptic trafficking (44). Protein ubiquitination has emerged as a key regulator of AMPAR trafficking. In cultured neurons, both GluA1 and GluA2 undergo activity-dependent ubiquitination following stimulation with AMPAR agonists or GABA_A_ receptor antagonists (45–47). GluA2/GluA3 complexes also undergo endocytosis and lysosomal degradation in brain ischemia (48). In this study, we demonstrated that MG132, a specific proteasome inhibitor that blocks the degradation of ubiquitinated proteins, reversed GluA3 protein degradation induced by either α2δ-1 coexpression or nerve injury. Furthermore, α2δ-1 coexpression or nerve injury caused a profound increase in ubiquitination of GluA3 in the spinal cord. The spinal cord ubiquitin-proteasome system plays an important role in nerve injury–induced pain hypersensitivity (38, 39). Our findings suggest that its inhibition may alleviate neuropathic pain, at least in part, by preventing GluA3 degradation in the spinal cord.

Using MS-based ubiquitinome analyses, we identified K861 on the C-terminus of GluA3 as a putative site of ubiquitination in the presence of α2δ-1. Subsequent analyses using point mutation and immunoblotting confirmed that K861 is crucial for α2δ-1–induced GluA3 degradation. Our identification of ubiquitination sites may not be comprehensive because ubiquitination may also occur on other residues, such as cysteine, serine, and threonine (49, 50). The incomplete prevention of GluA3 degradation by the K861 mutation may reflect the involvement of additional ubiquitination sites. Moreover, the intracellular C-terminus of AMPARs plays a critical role in interactions with intracellular proteins and serves as the primary site for various posttranslational modifications, including phosphorylation, *O*-GlcNAcylation, ubiquitination, acetylation, palmitoylation, and nitrosylation (51). Thus, crosstalk between GluA3 ubiquitination and other posttranslational modifications may also contribute to GluA3 stability and degradation. Our findings suggest that α2δ-1 may cause conformational changes in GluA3, exposing its C-terminal lysine residues for ubiquitination and degradation. Further studies are needed to identify the specific E2 ubiquitin–conjugating enzymes and E3 ligases involved in this process.

Our study also highlights the functional significance of GluA3 abundance in the spinal cord in neuropathic pain and synaptic CP-AMPARs caused by nerve injury. We showed that increasing GluA3 expression in the spinal cord using lentiviral vectors attenuated pain hypersensitivity and eliminated synaptic CP-AMPARs in the spinal dorsal horn caused by nerve injury. The increased prevalence of synaptic CP-AMPARs represents a key synaptic plasticity that strengthens excitatory glutamatergic transmission and amplifies nociceptive input from primary afferents to the spinal dorsal horn in neuropathic pain (11–13). The surface expression of AMPARs is intricately regulated through a balance of biosynthesis, subunit assembly, dendritic transport, receptor recycling, and degradation processes (4, 10). These mechanisms are dynamically controlled by AMPAR-interacting proteins and various posttranslational modifications occurring on their cytoplasmic domains. Given that GluA3 homomers tend to aggregate and impede membrane trafficking, GluA3 proteins have distinct propensities for forming GluA1/GluA2/GluA3 or GluA2/GluA3 heteromeric AMPARs (6, 7). In neuropathic pain, when GluA3 in the spinal cord is induced by α2δ-1 for degradation, it becomes unavailable for assembly with GluA2 to form GluA2/GluA3 heterotetramers. Concurrently, α2δ-1 disrupts the heteromeric assembly of GluA1 and GluA2 (13), thereby favoring the formation of GluA1 homotetrameric CP-AMPARs and their synaptic expression in neuropathic pain.

In this study, we found that lentiviral vector–mediated GluA3 expression at the spinal cord level increased GluA2/GluA3 heteromer formation in nerve-injured animals, but not in sham controls. This suggests that under normal conditions, GluA2 preferentially assembles with GluA1, forming GluA1/GluA2 heterotetramers that constitute the primary functional AMPARs at glutamatergic synapses. In contrast, nerve injury elevates α2δ-1 levels, which disrupts GluA1/GluA2 heteromeric assembly in the spinal dorsal horn, leading to the accumulation of unassembled GluA2 in the endoplasmic reticulum (13). Consequently, increased GluA3 protein, driven by *Gria3* gene delivery, preferentially pairs with excess GluA2 to form GluA2/GluA3 heteromers when α2δ-1 levels are elevated. This shift in AMPAR subunit composition — from GluA1 homomeric, CP-AMPARs to GluA2/GluA3 heteromeric, Ca^2+^-impermeable AMPARs — likely contributes to the reversal of nerve injury–induced pain hypersensitivity by GluA3 overexpression. Because the GluA3–α2δ-1 protein complex undergoes rapid proteasomal degradation, spinal cord GluA3 overexpression may also alleviate pain hypersensitivity by reducing α2δ-1 levels upregulated by nerve injury. Although intrathecal injection of *Gria3*-expressing vectors may induce GluA3 expression in the dorsal root ganglion, GluA3 alone tends to aggregate, which hinders its transport to the cell surface and results in minimal functional activity (6). Interestingly, we observed that GluA3 overexpression increased GluA2 protein levels in the spinal cord, although the underlying mechanism remains unclear. Notably, GluA2 levels are reduced in GluA3-KO mice (52), suggesting that GluA3 may act as a stabilizing factor for GluA2, indirectly contributing to elevated GluA2 levels by facilitating the assembly and/or trafficking of GluA2/GluA3 heteromers.

Our findings advance our holistic understanding of the molecular mechanism underlying the therapeutic actions of gabapentinoids in neuropathic pain. α2δ-1 is the primary target of gabapentinoids, which are used clinically to treat chronic neuropathic pain. Although α2δ-1 is commonly known as a voltage-activated Ca^2+^ channel subunit, gabapentinoids have no effects on Ca^2+^ channel activity or Ca^2+^ channel–mediated synaptic transmission (13, 22, 53–56). Gabapentinoids effectively inhibit both α2δ-1–bound NMDARs and α2δ-1–mediated CP-AMPARs in vitro and in vivo (13, 22, 25, 30, 31, 57–59). Although constitutive *Cacna2d1* KO produced only a small effect on the development of pain hypersensitivity after nerve injury, antisense-mediated *Cacna2d1* knockdown (24) or gabapentinoid treatment (22, 33, 60, 61) produces substantially greater inhibition of nerve injury–induced pain hypersensitivity. This disparity suggests that α2δ-1 may have a more prominent role in the maintenance rather than the initiation of chronic neuropathic pain. Alternatively, developmental compensation in constitutive *Cacna2d1*-KO mice may contribute to the attenuated effect on neuropathic pain development. In the present study, we found that pregabalin effectively reduced α2δ-1–induced GluA3 degradation and restored spinal GluA2/GluA3 heterotetramers reduced by nerve injury. Because GluA2/GluA3 receptors are typically in a low-conductance state (62), gabapentinoids could reduce neuropathic pain by promoting the assembly and synaptic expression of both GluA1/GluA2 and GluA2/GluA3 heterotetramers, thus displacing the high-conductance GluA1 homomeric receptors at synapses (5, 63). In line with our previous reports (13, 22, 59), α2δ-1 dynamically interacts with phosphorylated NMDARs and AMPARs through its C-terminus to (a) promote synaptic expression of NMDARs, (b) disrupt GluA1/GluA2 heteromeric assembly, and (c) induce GluA3 proteasomal degradation. Through these mechanisms, α2δ-1 augments intracellular Ca^2+^ levels and enhances glutamatergic transmission. Gabapentinoids collectively inhibit these α2δ-1 actions, diminishing synaptic NMDAR activity and restoring the assembly and postsynaptic dominance of GluA2-containing, Ca^2+^-impermeable AMPARs in the spinal cord in neuropathic pain. Gabapentinoids bind to both α2δ-1 and α2δ-2, with some adverse effects, such as ataxia, arising from the inhibition of α2δ-2–bound kainate receptors in the cerebellum (53). Notably, treatment with an α2δ-1 C-terminal peptide inhibits all α2δ-1–mediated actions on NMDARs and AMPARs, suggesting that targeting the C-terminal domain of α2δ-1 could offer an alternative treatment for chronic pain with potentially fewer adverse effects.

In conclusion, our study uncovers that α2δ-1 induces GluA3 degradation via the ubiquitin-proteasome pathway, representing another key molecular mechanism underlying the increased prevalence of synaptic CP-AMPARs in neuropathic pain (Supplemental Figure 7). This finding reinforces the role of α2δ-1 as the predominant regulator of glutamatergic synaptic plasticity, particularly in orchestrating AMPAR subunit assembly and composition, and enhances our understanding of the molecular mechanisms by which gabapentinoids alleviate neuropathic pain. Increasing spinal cord GluA3 levels may represent a potential strategy to reduce synaptic CP-AMPARs and alleviate neuropathic pain. CP-AMPARs are implicated in a range of neurological disorders, including drug abuse (14), epilepsy (64), ischemic stroke (65), Parkinson’s disease (66), and neurogenic hypertension (15, 17). Additionally, amyloid-β may induce synaptic and cognitive deficits in Alzheimer’s disease through GluA3-containing AMPARs (67). Therefore, elucidating the regulatory mechanisms of GluA3 protein homeostasis could inform the development of alternative therapeutic strategies for these disorders.

## Methods

### Sex as a biological variable.

Sex was not considered as a biological variable because no differences were observed in neuropathic pain behaviors or agent treatment effects between male and female animals in this study.

### Animal models.

For the generation of *Cacna2d1*-KO mice, 2 breeding pairs of *Cacna2d1*^+/–^ mice (C57BL/6 genetic background) were purchased from Medical Research Council (stock 6900). The generation of *Cacna2d1*-KO mice was previously reported (27). *Cacna2d1*^–/–^ and *Cacna2d1*^+/+^ (WT) littermates were obtained by breeding *Cacna2d1*^+/–^ mice.

To induce neuropathic pain in mice, SNI was performed on both male and female mice (10–11 weeks old) under 2% isoflurane anesthesia, as previously described (13, 68). The left common peroneal and tibial nerves were ligated and transected, while the sural nerve was left intact. The sham procedure followed the same procedure without nerve ligation or transection.

SNL was used to induce neuropathic pain in rats (12, 69). Male Sprague-Dawley rats (10–11 weeks old; Envigo) were anesthetized with 2% isoflurane, and the left L5 and L6 spinal nerves were isolated and ligated with a 6-0 silk suture. Sham surgery, in which the nerve was exposed but not ligated, served as the control.

Intrathecal catheters (PE-10 polyethylene tubing) were implanted in rats under isoflurane anesthesia (12, 20). The catheters were advanced 8 cm caudally through an incision in the cisternal membrane and secured to the musculature at the incision site. Rats were allowed to recover for 5–7 days before receiving intrathecal treatment.

### Nociceptive behavioral tests.

To measure the tactile withdrawal threshold, animals were placed in an individual plastic box on a mesh floor. A series of calibrated von Frey filaments were applied perpendicularly to the plantar surface of the hindpaw with sufficient force to bend the filaments for 6 seconds. A brisk paw withdrawal or flinch was considered a positive response. In the absence of a response, the next filament with greater force was applied. If a response occurred, the next filament with lower force was used. Six consecutive responses following the first change were used to calculate the withdrawal threshold (in grams) using the “up-down” method (70, 71).

The pressure withdrawal threshold was assessed using a Randall Selitto paw pressure device (catalog 2500, IITC Life Science). The device gently held the animal’s hindpaw while a steadily increasing force was applied via a pointed end to the midplantar glabrous surface. The force was immediately stopped upon a withdrawal response, and the threshold was recorded (26, 72).

The thermal withdrawal latency was measured with a thermal testing apparatus (catalog 390G, IITC Life Science). Animals were placed on a glass surface maintained at 30°C and allowed to acclimate to the device. A mobile radiant heat stimulus was then applied to the plantar surface of the hindpaw until the animal lifted or licked the hindpaw (57, 73). The time taken for hindpaw withdrawal was recorded as the withdrawal latency.

### Lentiviruses expressing Cacna2d1 and Gria3, and cell culture and transfection.

Detailed procedures for generating lentiviruses expressing *Cacna2d1* and *Gria3*, as well as for cell culture and transfection, are provided in the Supplemental Methods. The cDNAs used for α2δ, YFP-tagged WT α2δ-1, and α2δ chimeras have been described previously (13, 22).

### Electrophysiological recording in HEK293 cells.

Whole-cell voltage-clamp recordings were conducted using an EPC-10 amplifier (HEKA Instruments). The current-voltage relationship of glutamate-elicited currents was determined using a voltage ramp from –80 to 70 mV at 100 mV/s (13). The rectification index was calculated by dividing the current amplitude recorded at +50 mV by that at –50 mV. Current densities were calculated by using the current amplitudes recorded at –60 mV. The extracellular recording solution consisted of (in mM) 145 NaCl, 2.5 KCl, 1 CaCl_2_, 1 MgCl_2_, 10 glucose, and 10 HEPES (pH 7.3; osmolarity, 320 mOsm). Electrodes (resistance, 4–6 MΩ) were filled with a pipette solution containing (in mM) 145 CsCl, 2.5 NaCl, 10 HEPES, 1 EGTA, 4 MgATP, and 0.1 spermine tetrahydrochloride (pH 7.3, 300 mOsm). The cell membrane capacitance and series resistance were electronically compensated. Glutamate (10 mM; Sigma-Aldrich) and cyclothiazide (100 μM; Tocris Bioscience) were applied via a pressurized perfusion system (ALA Scientific Instruments) to elicit AMPAR currents.

### Electrophysiological recordings in spinal cord slices.

Rats were deeply anesthetized with 3% isoflurane, and the lumbar spinal cords were promptly removed through laminectomy. Transverse tissue slices (400 μm thick) were then prepared using a vibratome and submerged in sucrose-modified artificial cerebrospinal fluid saturated with 95% O_2_ and 5% CO_2_. The composition of the artificial cerebrospinal fluid was as follows (in mM): 234 sucrose, 26 NaHCO_3_, 3.6 KCl, 2.5 CaCl_2_, 1.2 MgCl_2_, 1.2 NaH_2_PO_4_, and 25 glucose. Subsequently, the slices were transferred to Krebs solution containing (in mM) 117 NaCl, 25 NaHCO_3_, 3.6 KCl, 2.5 CaCl_2_, 1.2 MgCl_2_, 1.2 NaH_2_PO_4_, and 11 glucose. All slices were incubated in a continuously oxygenated chamber for a minimum of 1 hour at 34°C before being utilized for recordings.

The spinal cord slices were carefully transferred into a recording chamber and perfused continuously with oxygenated Krebs solution at a rate of 3 mL/min at 34°C. Lamina II neurons were identified under an upright microscope equipped with infrared and differential interference contrast optics (BX51WI, Olympus Optical Co.). Glass recording electrodes (with resistance ranging from 5 to 8 MΩ) were filled with an internal solution containing (in mM) 135 potassium gluconate, 5 KCl, 2 MgCl_2_, 0.5 CaCl_2_, 5 ATP-Mg, 0.5 Na_2_-GTP, 5 EGTA, 5 HEPES, and 10 lidocaine *N*-ethyl bromide (pH 7.3, 280–300 mOsm). EPSCs were recorded in whole-cell voltage-clamp mode at a holding potential of –60 mV. Monosynaptic EPSCs were evoked by electrical stimulation (0.6 mA, 0.5 ms, and 0.1 Hz) of the ipsilateral dorsal root using a bipolar tungsten electrode. Monosynaptic EPSCs were identified by their consistent latency and the lack of conduction failure during 20-Hz stimulation (31, 74). Signal filtering was set at 1–2 kHz, and all signals were processed through a Multiclamp 700B amplifier (Molecular Devices) before being digitized at 20 kHz using a DigiData 1550B (Molecular Devices).

l-Glutamate (G1626) was purchased from MilliporeSigma, and cyclothiazide (catalog 0713) was purchased from Tocris Bioscience. Pregabalin (catalog 13663), IEM-1460 (catalog 121034-89-7), and MG132 (catalog 133407-82-6) were acquired from Cayman Chemical. α2δ-1CT peptide (VSGLNPSLWSIFGLQFILLWLVSGSRHYLW) and scrambled control peptide (FGLGWQPWSLSFYLVWSGLILSVLHLIRSN), both fused to the Tat domain (YGRKKRRQRRR), were synthesized by Synpeptide Co. and validated using liquid chromatography and MS.

### Immunoblotting and coimmunoprecipitation.

We collected protein samples from HEK293 cells and fresh dorsal spinal cord tissues at the L4–L6 levels in rats and at L3–L5 levels in mice. HEK293 cells were transfected with PolyJet DNA in vitro transfection reagent (catalog SL100688, SignaGen Laboratories). The transfection ratio of GluA2, GluA3, and α2δ-1 was maintained at 1:1:1 in most of the experiments. However, when testing the effects of α2δ-1CT peptides (1 μM), pregabalin (20 μM), and MG132 (10 μM), as well as the GluA3 gene mutants, we reduced the ratio of α2δ-1/GluA3 plasmids to 1:10 to reduce excessive GluA3 degradation. In experiments assessing GluA3 ubiquitination, we transfected GluA3 plasmids in the control group at one-third of the amount used in the α2δ-1/GluA3 coexpression group to equalize GluA3 protein levels between groups. Additionally, we reduced the amount of α2δ-1 to one-fifth of GluA3 to ensure sufficient GluA3 for the pull-down assay. Empty vectors were used to equalize the total DNA amount across transfection groups.

In coimmunoprecipitation assays, we collected protein samples from transfected HEK293 cells and spinal cord tissues. The rabbit anti-GluA3 antibody (mAb5117, Cell Signaling Technology) or rabbit anti-GluA2 antibody (AB10529, EMD Millipore) was preincubated with protein G agarose beads at 25°C for 1 h, and the protein samples were then exposed to the antibody-conjugated beads at 4°C overnight. After the pulldown, the protein samples were subjected to gel electrophoresis using 4% SDS for immunoblotting analysis. Ubiquitin, GluA2, and GluA3 proteins were probed using mouse antiubiquitin (1:1,000; sc-8017, Santa Cruz Biotechnology), mouse anti-GluA2 (1:1,000; 75-002, NeuroMab), and mouse anti-GluA3 (1:1,000; MAB5416, MilliporeSigma) antibodies, followed by an HRP-linked anti-mouse secondary antibody (1:10,000; 7076, Cell Signaling Technology).

For other immunoblotting assays, we used the following primary antibodies: rabbit anti-GluA3 (1:1,000; mAb5117, Cell Signaling Technology), rabbit anti-GFP (1:1,000; mAb2555, Cell Signaling Technology), rabbit anti-HA tag (1:1,000; mAb3724, Cell Signaling Technology), mouse anti–α2δ-1 (1:1,000; sc-271697, Santa Cruz Biotechnology), rabbit anti-GAPDH (1:5,000; mAB2118, Cell Signaling Technology), and mouse anti–β-actin (1:10,000; mAb3700, Cell Signaling Technology). HRP-linked anti-rabbit (1:10,000; 7074, Cell Signaling Technology) and anti-mouse (1:10,000; 7076, Cell Signaling Technology) secondary antibodies were used. Immunoblotting data were collected and quantified using LI-COR Image Studio software (LI-COR Biosciences). For protein quantification, the intensity of the protein bands was normalized to housekeeping protein GAPDH or β-actin.

### Synaptosome preparation.

The dorsal spinal cord tissues at L4–L6 levels were homogenized using a Dounce tissue grinder (Sigma-Aldrich). Synaptic proteins were extracted and enriched using Syn-PER reagent (Thermo Fisher Scientific) containing a protease inhibitor cocktail (MilliporeSigma). The homogenate was centrifuged at 1,200*g* for 10 minutes at 4°C to remove the nuclei and large debris. The supernatant was then centrifuged at 15,000*g* for 20 minutes to obtain the crude synaptosomes. The synaptosomal pellets were incubated in RIPA lysis buffer with a protease inhibitor cocktail for 1 h on ice and then centrifuged at 16,000*g* for 15 minutes at 4°C to obtain the synaptosomal proteins for immunoblotting analysis. Synaptosomal proteins were probed with rabbit anti-GluA3 (1:1,000; 5117, Cell Signaling Technology), mouse anti–α2δ-1 (1:1,000; sc-271697, Santa Cruz Biotechnology), or mouse anti–PSD-95 (1:10,000; MABN1190, MilliporeSigma) antibodies, followed by HRP-linked anti-rabbit (1:10,000; 7074, Cell Signaling Technology) or anti-mouse (1:10,000; 7076, Cell Signaling Technology) secondary antibodies. The intensity of the protein bands was normalized to PSD-95 on the same gel.

### Immunofluorescence labeling.

Rats were deeply anesthetized with phenytoin/pentobarbital solution (0.1 mL/kg, i.p.) and then transcardially perfused with 4% paraformaldehyde in 0.1 M PBS. The spinal cords were dissected and postfixed for 2 h with the same fixative, immersed in 30% sucrose solutions in PBS for 24–48 h, and subsequently frozen in Tissue-Tek optimal cutting temperature compound (Sakura Finetek USA). Sections of the spinal cords were cut to a thickness of 30 μm and collected free-floating in 0.1 M Tris buffer (TBS). Prior to antibody incubation, the sections were rinsed in 0.1 M TBS for 30 minutes, quenched with 3% hydrogen peroxide in TBS for 30 minutes, and blocked with TNB (0.5% block reagent in 0.1 M TBS) for 60 minutes at 24°C. For double labeling of GluA3 and NeuN, sections were incubated in a mixture of primary antibodies: rabbit anti-GluA3 (1:50; mAb5117, Cell Signaling Technology) and mouse anti-NeuN (1:300; ab104224, Abcam), diluted in TNB solution for 2 h at 24°C and then for 24 h at 4°C. On the following day, all sections were rinsed in 0.1 M TBS for 30 minutes and then incubated with the secondary antibody, horseradish peroxidase–conjugated goat anti-rabbit (1:300; 111-035-144, Jackson ImmunoResearch) for 1 h at 24°C, followed by rinsing with TBS for 30 minutes. Subsequently, the sections were incubated with FITC-tyramide (1:100; NEL701, PerkinElmer) for 10 minutes. After rinsing for 30 minutes, the sections were further incubated with Alexa Fluor 647 conjugated to goat anti-mouse IgG (1:200; A31571, Molecular Probes) for 60 minutes. For IB4 labeling, sections were rinsed and incubated with IB4 conjugated to Alexa Fluor 594 (2 μg/mL; I-21413, Molecular Probes) for 2 h. Subsequently, sections were rinsed again with 0.1 M TBS, mounted on slides, dried, and coverslipped.

For GluA3 immunofluorescence labeling in the cell line, HEK293 cells were transfected with GluA3 or GluA3 plus α2δ-1–GFP at a transfection ratio of 1:1 for GluA3/α2δ-1–GFP. The cells were plated on poly-d-lysine–coated coverslips and cultured overnight before the immunofluorescence experiment. For fixation, we used 4% paraformaldehyde (Millipore Sigma) for 10 minutes at 24°C. After a brief rinse, the cells were blocked with 4% normal goat serum (Vector Laboratories), followed by immunolabeling with a rabbit anti-GluA3 antibody (1:100; 5117, Cell Signaling Technology) overnight at 4°C. A goat anti-rabbit IgG-Alexa Fluor 594 secondary antibody (Invitrogen Life Technologies) was then added and incubated at 24°C for 90 minutes. Finally, the slides were washed, dried, and mounted. Slides were examined on a laser scanning confocal microscope (LSM510, Zeiss), and regions of interest were photodocumented with a 25× oil immersion objective (numerical aperture 0.8). Confocal images were processed using the Zeiss LSM Image Browser (version 3.5.0.359).

### Ubiquitination assay.

The MS-based ubiquitination assay was conducted on GluA3 transiently coexpressed with α2δ-1 in HEK293 cells. The transfection ratio between Flag-tagged GluA3 and α2δ-1 was set at 5:1 to ensure sufficient GluA3 proteins remained available for the MS assay. MG132 (10 μM) was applied overnight before sample harvesting. After 48 h of transfection, the cells were collected, and Flag-tagged GluA3 was enriched through immunoprecipitation using a rabbit anti-Flag antibody (F7425, MilliporeSigma). The immunoprecipitated proteins were separated by SDS-PAGE and visualized with Coomassie blue G-250 (Thermo Fisher Scientific). Protein-containing gel bands were excised into 3 to 5 distinct sections. The excised bands were thoroughly washed with 50% acetonitrile in 25 mM ammonium bicarbonate and then subjected to in-gel reduction with dithiothreitol, alkylation with iodoacetamide, and digestion with trypsin (V5280, Promega), as previously reported (59, 75). Digested peptide mixtures were extracted, dried in a speed vacuum concentrator, and reconstituted into 2% acetonitrile and 0.1% trifluoroethanol. MS was performed with an Orbitrap Fusion Lumos Tribrid mass spectrometer (Thermo Fisher Scientific) coupled to an Ultimate 3000 RSLC-Nano liquid chromatography system. Samples were injected onto a 75 μm i.d., 75 cm long EasySpray column (Thermo Fisher Scientific) and eluted with a gradient from 0 to 28% buffer B over 90 minutes. Buffer A contained 2% (v/v) acetonitrile and 0.1% (v/v) formic acid in water, and buffer B contained 80% (v/v) acetonitrile, 10% (v/v) trifluoroethanol, and 0.1% formic acid in water. The mass spectrometer was operated in positive ion mode with a source voltage of 1.5–2.4 kV and an ion transfer tube temperature of 275°C. MS scans were acquired in the Orbitrap, and up to 10 MS/MS spectra were obtained in the ion trap (0.6 Da mass resolution) for each full spectrum acquired using higher-energy collisional dissociation for ions with charges of 2–7. Dynamic exclusion was set for 25 seconds after an ion was selected for fragmentation. The spectra labeling, database search, and quantification were carried out with Mascot Distiller (v2.6 or 2.7) and Mascot (v2.4 or 2.7) (MatrixScience). MS/MS spectra were searched against the human Uniprot Proteomes database (release 2020_09, 75,069 protein entries; https://ftp.uniprot.org/pub/databases/uniprot/previous_releases/) supplemented with rat GluA3 and α2δ-1 protein sequences. Database searches were conducted using a peptide mass tolerance of 20 ppm and an MS/MS fragment ion tolerance of 0.5–0.6 Da, allowing up to 2 missed cleavage sites and considering variable modifications including carbamidomethylation (C), oxidation (M), N-terminal pyroglutamate (pyro-Glu), and diglycine (GlyGly) on lysine residues (K). Given that trypsin cleavage can occur commonly at ubiquitinated Lys (76), peptides with a putative diglycine-modified Lys positioned at the C-terminus were not excluded in this study. MS/MS spectra indicative of potential ubiquitination were manually checked. Annotated MS/MS spectra were generated by labeling only the peptide matches used for scoring with Mascot (v2.7). The raw MS/MS data generated in this study have been deposited in the MassIVE database (https://massive.ucsd.edu/ProteoSAFe/dataset.jsp?task=3544d97b3de348468224f992034727d1; accession code MSV000098625).

### Mutagenesis and plasmid constructs.

Point mutations were introduced by replacing lysine with arginine at sites K710, K861, and K887 on rat GluA3 cDNA. The mutagenesis primers were designed using the In-Fusion Cloning Primer design tool, which is available on the Takara Bio website. The Flag-tagged GluA3 construct was created by inserting a Flag peptide (DYKDDDDK) sequence at the N-terminus of GluA3, using the following primers: forward, ACAAAGACGATGACGACAAGGGATTCCCCAACACCATCAGC; reverse, CGTCATCGTCTTTGTAGTCTCCGTGAGAATGACCCAAAAGC. The HA-tagged α2δ-1 construct was created by inserting an HA peptide (YPYDVPDYA) sequence at the N-terminus of α2δ-1, using the following primers: forward, ATACGATGTTCCAGATTACGCTGAGCCCTTCCCTTCGCCC; reverse, TCTGGAACATCGTATGGGTACTCGCTCGAGGGGCCGATC. HA-tagged α2δ-2 (plasmid 58731) and α2δ-3 (plasmid 58728) constructs were obtained from Addgene. All mutagenesis and DNA constructs were made using the In-Fusion HD Cloning Kit (Takara Bio) following the manufacturer’s instructions. The accuracy of all mutations and DNA constructs was confirmed through DNA sequencing.

### Statistics.

All data are presented as means ± SEM. The sample sizes were consistent with those typically employed in the field (13, 22, 59). Animals were assigned to the control and treatment groups with 1:1 allocation based on availability. No animal deaths occurred during the final experiments, and no outlier tests were performed. Data from male and female mice were pooled, as no sex differences were observed in the behavioral and biochemical data. Investigators performing behavioral tests and electrophysiological recordings were blinded to treatment groups. AMPAR current data in HEK293 cells were analyzed using Pulse software (HEKA Instruments), and the current density was calculated by normalizing the current to capacitance to adjust for variations in cell sizes. The rectification index was calculated using a voltage ramp from –80 to 70 mV at 100 mV/s during bath application of glutamate. The amplitude of evoked EPSCs was analyzed using Clampfit 10.0 software (Molecular Devices). Immunoblotting data were quantified using LI-COR Image Studio software (LI-COR Biosciences). Normality of datasets was evaluated using the Shapiro-Wilk test. We used 2-tailed Student’s *t* test to compare 2 groups. To compare more than 2 groups, we used 1- or 2-way ANOVA followed by Dunnett’s or Tukey’s post hoc test. Statistical analyses were performed using Prism software (version 10, GraphPad), and differences were considered to be statistically significant if the *P* value was less than 0.05.

### Study approval.

All experimental procedures were approved (approval 1231-RN02 and 0660-RN03) by the Institutional Animal Care and Use Committee of The University of Texas MD Anderson Cancer Center and adhered to NIH guidelines on the ethical use of animals.

### Data availability.

Values for all data points in figures are reported in the Supporting Data Values file. Data generated in this study are available from the corresponding author upon reasonable request.

## Author contributions

MHZ, SRC, DJ, YH, and HC performed research. MHZ, SRC, DJ, GC, and JY analyzed data. MHZ and HLP drafted the manuscript. HLP designed research and finalized the manuscript, with input from the other authors.

## Funding support

This work is the result of NIH funding, in whole or in part, and is subject to the NIH Public Access Policy. Through acceptance of this federal funding, the NIH has been given a right to make the work publicly available in PubMed Central.

- NIH grants NS101880 and NS132398 to HLP and SRC.
- Pamela and Wayne Garrison Distinguished Chair Endowment to HLP.

## Supplementary Material

Supplemental data

Unedited blot and gel images

Supporting data values

## Figures and Tables

**Table 1:**
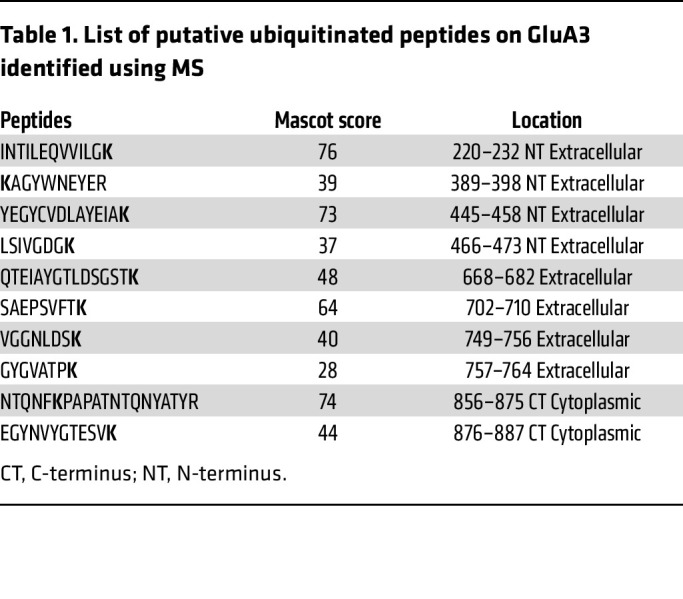
List of putative ubiquitinated peptides on GluA3 identified using MS

## Version 1 10/23/2025

In-Press Preview

## Version 2 01/02/2026

Electronic publication
