# Supplementary material for: Spinal α2δ-1 induces GluA3 degradation to regulate assembly of calcium-permeable AMPA receptors and pain hypersensitivity
Source: J Clin Invest. 2025 Oct 23;136(1):e193349. doi: 10.1172/JCI193349 (PMC12721899; doi:10.1172/JCI193349)
Supplement: Supplemental data [file jci-136-193349-s101.pdf]

Supplemental Methods

Generation of lentiviruses expressing *Cacna2d1* and *Gria3*

We constructed lentiviruses expressing the full-length rat *Cacna2d1* (Sequence ID: NM_012919) and rat *Gria3* (Sequence ID: NM_032990.2) coding sequences. Briefly, we performed PCR using CloneAMP HiFi PCR premix (#639298, TaKara) to obtain the full-length cDNA of *Cacna2d1* from the mRNA of rat spinal cord. The *Cacna2d1* cDNA was then cloned into a pEntry vector using the pEntry Directional Topo Cloning kit (#K2400-20, Invitrogen). The *Cacna2d1* in the pEntry vector was recombined into pLenti7.3 based on the instruction in the pLenti7.3/V5 Gateway Vector Kit (#V534-06, Invitrogen). The pLenti7.3/V5 vector containing *Cacna2d1* was then packaged into the lentivirus through co-transfecting with the packaging plasmids pCMV-VSV-G (#8454, Addgene) and psPAX2 (#12260, Addgene) into HEK293FT cells. The virus-containing culture medium was collected after 72 h of culture. The virus was concentrated 100 times using a Lenti-X concentrator reagent (#631231, Takara Bio), and the viral titer was determined using a titration reagent (#VPK-112, Cell Biolabs). The concentrated virus with a titer of 4×10^{11} viral particles/mL was stored at –80 °C until use. The negative control virus was made using pLenti7.3/V5 vector (without *Cacna2d1*) with the same packaging method. Same methods are used for the construction of lentiviruses expressing *Gria3*. The titer of the concentrated *Gria3* lentivirus is 2.5×10^{11} viral particles/mL. The primers used for PCR are *Cacna2d1*-forward: 5'-CACCatggctgctggctgcctgctgg-3'; *Cacna2d1*-reverse: 5'-gtcaccatagatagtgtctgc-3'; *Gria3*-forward:5'-CACCatggggcaaagcgtgctccgggcggtc-3'; *Gria3*-reverse: 5'-tccctagatcttaacactttctgttcc-3'. The CACC sequence in the forward primers provides overhang base pairs, allowing for insertion into the pEntry vector.

Cell culture and transfection

HEK293 cells were cultured in Dulbecco's Modified Eagle's Medium (DMEM; Gibco/Life Technologies) supplemented with 10% fetal bovine serum (FBS; Sigma-Aldrich) at 37°C in a 5% CO_2 incubator. For electrophysiological recordings, 1.2×10^4 cells were plated on poly-D-lysine-coated coverslips. DNA encoding GluA2(R) or GluA3 was transfected along with either empty vectors (pcDNA) or α2δ subunits. Empty vectors were used to keep the total DNA amount balance across different transfection groups. The transfection ratio of GluA2, GluA3, and α2δ-1 was maintained at 1:1:1 in most experiments using the PolyJet DNA In Vitro Transfection Reagent (SignaGen Laboratories). GFP coexpression was used to identify transfected cells. Electrophysiological recordings were conducted 48 h post-transfection. The cDNAs used for α2δ, YFP-tagged WT α2δ-1, and α2δ chimeras have been previously described (13, 22). The GluA2(R) and GluA3 constructs were generously provided by Dr. Michael Hollmann (Ruhr University) and Dr. Li Niu (University at Albany), respectively.

Supplemental Figures

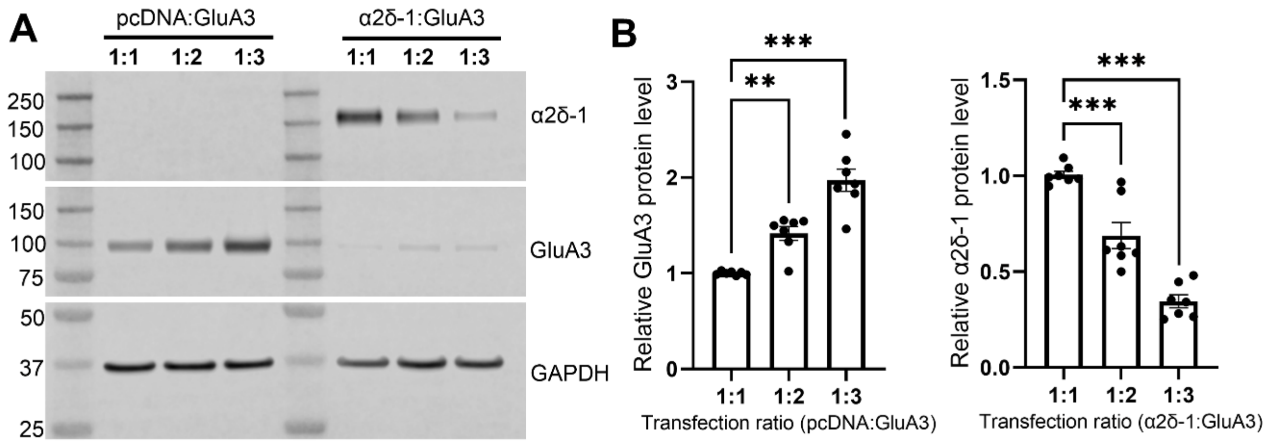

Figure S1. GluA3 overexpression reduces α2δ-1 protein levels. (**A** and **B**) Representative immunoblot images (**A**) and quantification (**B**) show the effects of coexpression with varying amounts of GluA3 on α2δ-1 protein levels in HEK293 cells. Empty vectors (pcDNA) were used to equalize the total DNA amount across different transfection groups (n = 7 independent experiments per group). **p < 0.01, ***p < 0.001; one-way ANOVA followed by Dunnett's *post hoc* test. Data are expressed as means ± SEM.

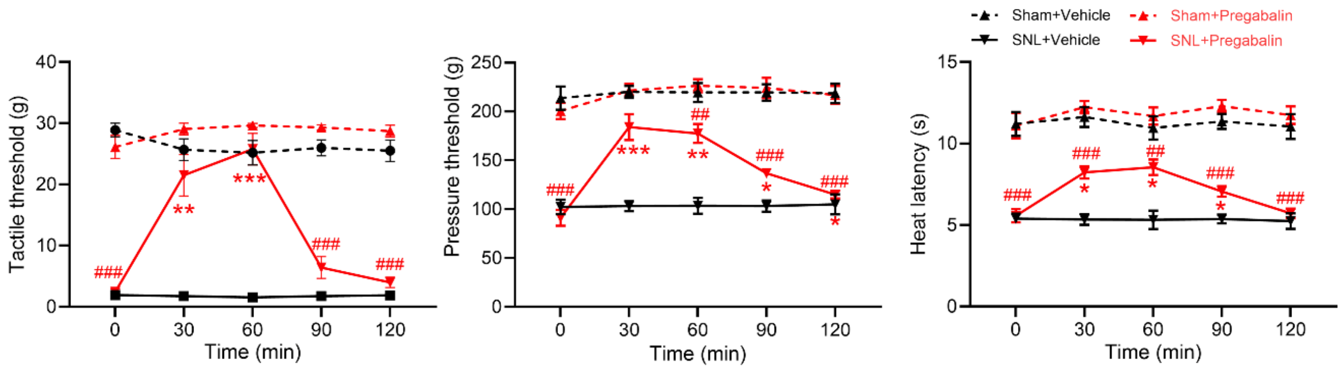

Figure S2. Pregabalin reverses nociceptive hypersensitivity induced by nerve injury. Time-course effects of intrathecal injection of pregabalin (10 µg) or vehicle on tactile, pressure, and heat withdrawal thresholds in sham and SNL rats 3 weeks after surgery (n = 7 rats per group). *p < 0.05, **p < 0.01, ***p < 0.001 vs. baseline (time 0); $^{\#\#}$p < 0.01, $^{\#\#\#}$p < 0.001 vs. SNL+Vehicle group at the same time point (two-way ANOVA followed by Tukey's *post hoc* test). Data are expressed as means ± SEM.

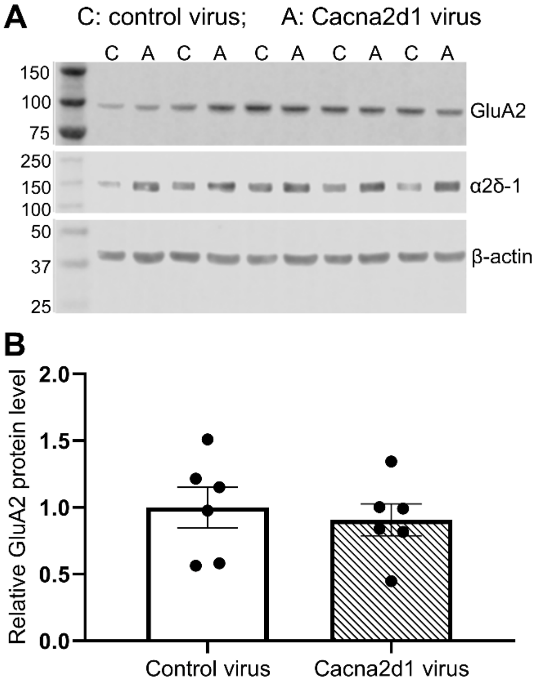

Figure S3. Effect of α2δ-1 overexpression on GluA2 protein levels in the spinal cord. (**A** and **B**) Representative immunoblot images (**A**) and quantification (**B**) show GluA2 protein levels in the dorsal spinal cord from naïve rats injected intrathecally with control lentiviruses or *Cacna2d1*-expressing lentiviruses (n = 6 rats per group). Two-tailed Student's t test. Data are expressed as means ± SEM.

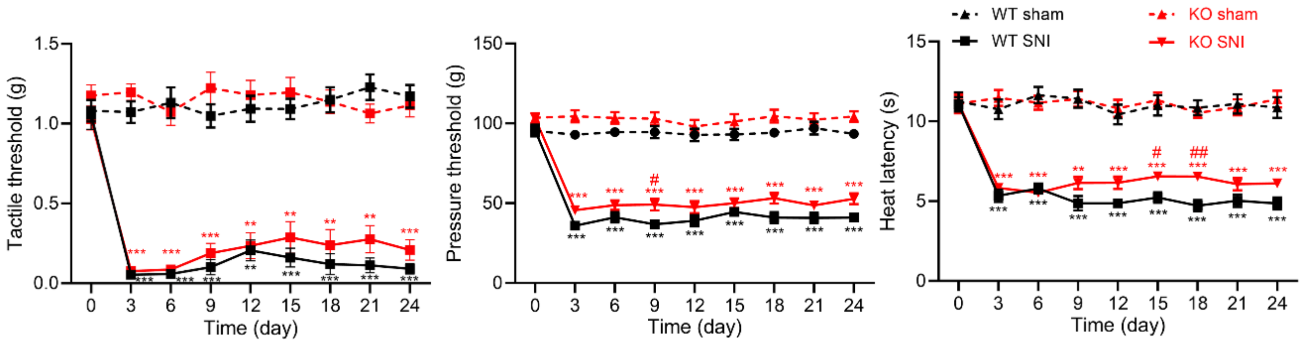

Figure S4. Development of nociceptive hypersensitivity after nerve injury in wild-type and *Cacna2d1* knockout mice. Time course of changes in tactile, pressure, and heat withdrawal thresholds in wild-type (WT) and *Cacna2d1* knockout (KO) mice after sham or SNI surgery (n = 10 rats per group). $^{**}p < 0.01$, $^{***}p < 0.001$ vs. baseline (day 0). $^{\#}p < 0.05$, $^{\#\#}p < 0.01$ vs. WT-SNI at the same time point (two-way ANOVA followed by Tukey's *post hoc* test). Data are expressed as means ± SEM.

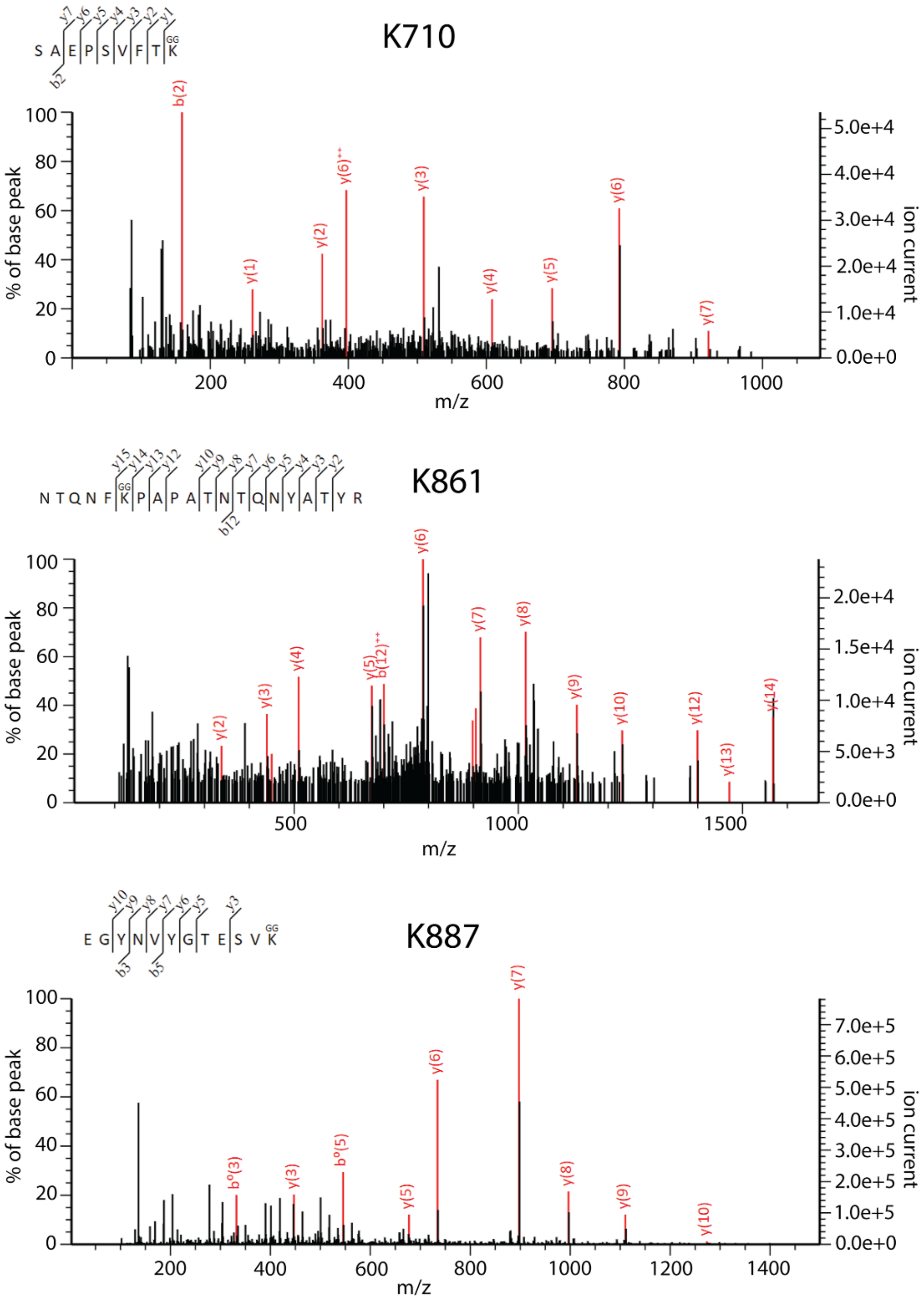

Figure S5. Annotated spectra of fragmented ions (tandem mass spectrometry) of putative GluA3 peptides including ubiquitination sites. The ubiquitination sites and peptide sequences are indicated on the top. Only b and y ions whose matches were qualified and used for scoring by Mascot version 2.7 are labeled. Annotation: b, B-ion; y, y-ion; number, position of fragmentation; m/z, mass-to-charge ratio; ^{O} loss of a water; $^{++}$ double charge; GG, diglycine-modified peptides.

```
CLUSTAL O(1.2.4) multiple sequence alignment


sp|P19490|GRIA1_RAT      ----MPYIFAFFCTGFLGAVVGANFPNNIQIGGLFPNQQSQEHAAFRFALSQLT------   50
sp|P19491|GRIA2_RAT      -MQKIMHISVLLSPVLWGLI-FGVSSNSIQIGGLFPRGADQEYSAFRVGMVQFST-----   53
sp|P19492|GRIA3_RAT      MGQSVLRAVFFLVLGLLGHS-HGGFPNTISIGGLFMRNTVQEHSAFRFAVQLYNTNQNTT   59
                             :     ::   : *    .   *.*.***** .   **::***..:   .

sp|P19490|GRIA1_RAT      -EPPKLLPQIDIVNISDSFEMTYRFCSQFSKGVYAIFGFYERRTVNMLTSFCGALHVCFI   109
sp|P19491|GRIA2_RAT      -SEFRLTPHIDNLEVANSFAVTNAFCSQFSRGVYAIFGFYDKKSVNTITSFCGTLHVSFI   112
sp|P19492|GRIA3_RAT      EKPFHLNYHVDHLDSSNSFSVTNAFCSQFSRGVYAIFGFYDQMSMNTLTSFCGALHTSFV   119
                          .  :*  ::* :: ::** :*  ******:*********:: ::* :*****:**..*:

sp|P19490|GRIA1_RAT      TPSFPVDTSNQFVLQLRPELQEALISIIDHYKWQTFVYIYDADRGLSVLQRVLDTAAEKN   169
sp|P19491|GRIA2_RAT      TPSFPTDGTHPFVIQMRPDLKGALLSLIEYYQWDKFAYLYDSDRGLSTLQAVLDSAAEKK   172
sp|P19492|GRIA3_RAT      TPSFPTDADVQFVIQMRPALKGAILSLLSYYKWEKFVYLYDTERGFSVLQAIMEAAVQNN   179
                         *****.*    **:*:** *: *::*::.:*:*:.*.*:**::**:*.** ::::*.:::

sp|P19490|GRIA1_RAT      WQVTAVNILTT----TEEGYRMLFQDLEKKKERLVVVDCESERLNAILGQIVKLEKNGIG   225
sp|P19491|GRIA2_RAT      WQVTAINVGNINNDKKDETYRSLFQDLELKKERRVILDCERDKVNDIVDQVITIGKHVKG   232
sp|P19492|GRIA3_RAT      WQVTARSVGNIKD---VQEFRRIIEEMDRRQEKRYLIDCEVERINTILEQVVILGKHSRG   236
                         ***** .: .       : :* :::::: ::*:  ::*** :::* *: *:: : *:  *

sp|P19490|GRIA1_RAT      YHYILANLGFMDIDLNKFKESGANVTGFQLVNYTDTIPARIMQQWRTSDSRDHTRVDWKR   285
sp|P19491|GRIA2_RAT      YHYIIANLGFTDGDLLKIQFGGANVSGFQIVDYDDSLVSKFIERWSTLEEKEYPGAHTAT   292
sp|P19492|GRIA3_RAT      YHYMLANLGFTDILLERVMHGGANITGFQIVNNENPMVQQFIQRWVRLDEREFPEAKNAP   296
                         ***::***** *  * :.  .***::***:*:  : :  :::::*   :.::.  ..

sp|P19490|GRIA1_RAT      PKYTSALTYDGVKVMAEAFQSLRRQRIDISRRGNAGDCLANPAVPWGQGIDIQRALQQVR   345
sp|P19491|GRIA2_RAT      IKYTSALTYDAVQVMTEAFRNLRKQRIEISRRGNAGDCLANPAVPWGQGVEIERALKQVQ   352
sp|P19492|GRIA3_RAT      LKYTSALTHDAILVIAEAFRYLRRQRVDVSRRGSAGDCLANPAVPWSQGIDIERALKMVQ   356
                          *******:*.: *::***: **:**:::****.************.**::*:***: *:

sp|P19490|GRIA1_RAT      FEGLTGNVQFNEKGRRTNYTLHVIEMKHDGIRKIGYWNEDDKFVPAATDAQAGGDNSSVQ   405
sp|P19491|GRIA2_RAT      VEGLSGNIKFDQNGKRINYTINIMELKTNGPRKIGYWSEVDKMVVTLTELPSGNDTSGLE   412
sp|P19492|GRIA3_RAT      VQGMTGNIQFDTYGRRTNYTIDVYEMKVSGSRKAGYWNEYERFVP-FSDQQISNDSSSSE   415
                         .:*::**::*:  *:* ***:.: *:* .* ** ***.* :::*   ::   ..*.*. :

sp|P19490|GRIA1_RAT      NRTYIVTTILEDPYVMLKKNANQFEGNDRYEGYCVELAAEIAKHVGYSYRLEIVSDGKYG   465
sp|P19491|GRIA2_RAT      NKTVVVTTILESPYVMMKKNHEMLEGNERYEGYCVDLAAEIAKHCGFKYKLTIVGDGKYG   472
sp|P19492|GRIA3_RAT      NRTIVVTTILESPYVMYKKNHEQLEGNERYEGYCVDLAYEIAKHVRIKYKLSIVGDGKYG   475
                         *:* :******.**** *** : :***:*******:** *****   .*:* **.*****

sp|P19490|GRIA1_RAT      ARDPDTKAWNGMVGELVYGRADVAVAPLTITLVREEVIDFSKPFMSLGISIMIKKPQKSK   525
sp|P19491|GRIA2_RAT      ARDADTKIWNGMVGELVYGKADIAIAPLTITLVREEVIDFSKPFMSLGISIMIKKPQKSK   532
sp|P19492|GRIA3_RAT      ARDPETKIWNGMVGELVYGRADIAVAPLTITLVREEVIDFSKPFMSLGISIMIKKPQKSK   535
                         *** :** ***********:**:*:***********************************

sp|P19490|GRIA1_RAT      PGVFSFLDPLAYEIWMCIVFAYIGVSVVLFLVSRFSPYEWHSEEFEEGRD--QTTSDQSN   583
sp|P19491|GRIA2_RAT      PGVFSFLDPLAYEIWMCIVFAYIGVSVVLFLVSRFSPYEWHTEEFEDGRE--TQSSESTN   590
sp|P19492|GRIA3_RAT      PGVFSFLDPLAYEIWMCIVFAYIGVSVVLFLVSRFSPYEWHLEDNNEEPRDPQSPPDPPN   595
                         ***************************************** *: ::         :  *

sp|P19490|GRIA1_RAT      EFGIFNSLWFSLGAFMQQGCDISPRSLSGRIVGGVWWFFTLIIISSYTANLAAFLTVERM   643
sp|P19491|GRIA2_RAT      EFGIFNSLWFSLGAFMQQGCDISPRSLSGRIVGGVWWFFTLIIISSYTANLAAFLTVERM   650
sp|P19492|GRIA3_RAT      EFGIFNSLWFSLGAFMQQGCDISPRSLSGRIVGGVWWFFTLIIISSYTANLAAFLTVERM   655
                         ************************************************************

sp|P19490|GRIA1_RAT      VSPIESAEDLAKQTEIAYGTLEAGSTKEFFRRSKIAVFEKMWTYMKSAEPSVFVRTTEEG   703
sp|P19491|GRIA2_RAT      VSPIESAEDLSKQTEIAYGTLDSGSTKEFFRRSKIAVFDKMWTYMRSAEPSVFVRTTAEG   710
sp|P19492|GRIA3_RAT      VSPIESAEDLAKQTEIAYGTLDSGSTKEFFRRSKIAVYEKMWSYMKSAEPSVFTKTTADG   715
                         **********:**********::**************::***:**:*******.:** :*

sp|P19490|GRIA1_RAT      MIRVRKSKGKYAYLLESTMNEYIEQRKPCDTMKVGGNLDSKGYGIATPKGSALRNPVNLA   763
```

```
sp|P19491|GRIA2_RAT      VARVRKSKGKYAYLLESTMNEYIEQRKPCDTMKVGGNLDSKGYGIATPKGSSLGNAVNLA   770
sp|P19492|GRIA3_RAT      VARVRKSKGKFAFLLESTMNEYIEQRKPCDTMKVGGNLDSKGYGVATPKGSALGNAVNLA   775
                         : ********:*:*******************************:******:* * ****

sp|P19490|GRIA1_RAT      VLKLNEQGLLDKLKNKWWYDKGECGSGGGDSKDKTSALSLSNVAGVFYILIGGLGLAMLV   823
sp|P19491|GRIA2_RAT      VLKLNEQGLLDKLKNKWWYDKGECGSGGGDSKEKTSALSLSNVAGVFYILVGGLGLAMLV   830
sp|P19492|GRIA3_RAT      VLKLNEQGLLDKLKNKWWYDKGECGSGGGDSKDKTSALSLSNVAGVFYILVGGLGLAMMV   835
                         ********************************:*****************:*******:*

sp|P19490|GRIA1_RAT      ALIEFCYKSRSESKRMKGFCLIPQQSINEAIRTSTLPRNSGAGASGGGGSGENGRVVSQD   883
sp|P19491|GRIA2_RAT      ALIEFCYKSRAEAKRMKVAKNPQ--NINPSSSQN--SQNFATYKEGYNVYGIESVKI---   883
sp|P19492|GRIA3_RAT      ALIEFCYKSRAESKRMKLTKNTQ--NFKPAPATN--TQNYATYREGYNVYGTESVKI---   888
                         **********:*:****        .:: :   .   :* .:  .* .  * :.  :

sp|P19490|GRIA1_RAT      FPKSMQSIPCMSHSSGMPLGATGL        907
sp|P19491|GRIA2_RAT      ------------------------        883
sp|P19492|GRIA3_RAT      ------------------------        888
```

Figure S6. Alignment of rat GluA1, GluA2, and GluA3 protein sequences. Putative ubiquitously modified peptides on GluA3, identified through a ubiquitinomic assay, are highlighted. Protein sequences and alignment were performed using UniProt databases.

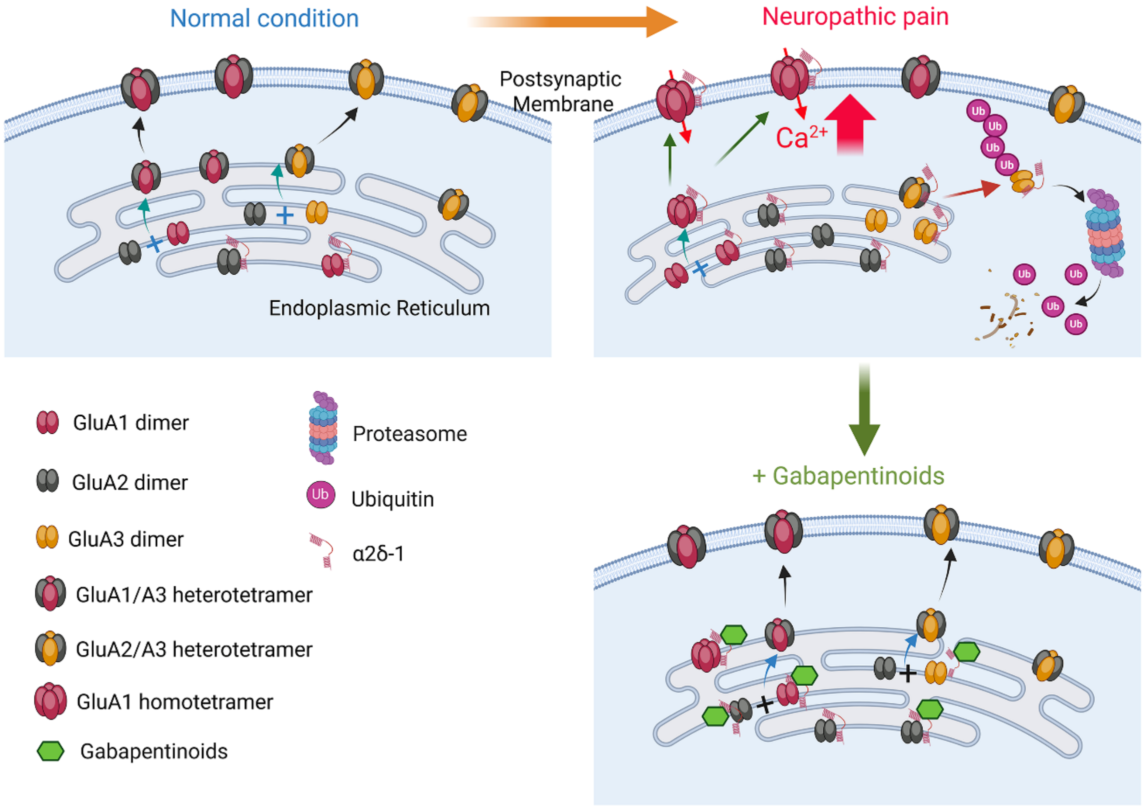

Figure S7. Graphical representations illustrating how α2δ-1 facilitates the assembly and surface expression of CP-AMPARs at spinal cord synapses, along with associated gabapentinoid actions in neuropathic pain. Under normal conditions, α2δ-1 has minimal interaction with GluA1, GluA2, or GluA3 in the spinal dorsal horn. GluA1/GluA2 and GluA2/GluA3 readily form Ca^{2+}-impermeable, heterotetrameric AMPARs for postsynaptic expression. In neuropathic pain conditions, α2δ-1 expression increases and directly interacts with GluA1, GluA2, and GluA3. This interaction not only disrupts GluA1/GluA2 heteromeric assembly but also induces ubiquitination of GluA3, leading to its degradation in proteasomes. As a result of these two actions, α2δ-1 promotes the assembly and synaptic expression of GluA1 homotetramers, resulting in elevated Ca^{2+} levels at spinal synapses. Gabapentinoids counteract these effects by inhibiting α2δ-1's actions, thereby restoring the heteromeric assembly and synaptic expression of GluA1/GluA2 and GluA2/GluA3.
