# Supplementary material for: Spinal α2δ-1 induces GluA3 degradation to regulate assembly of calcium-permeable AMPA receptors and pain hypersensitivity
Source: J Clin Invest. 2025 Oct 23;136(1):e193349. doi: 10.1172/JCI193349 (PMC12721899; doi:10.1172/JCI193349)

Full unedited blot for Fig. 2A

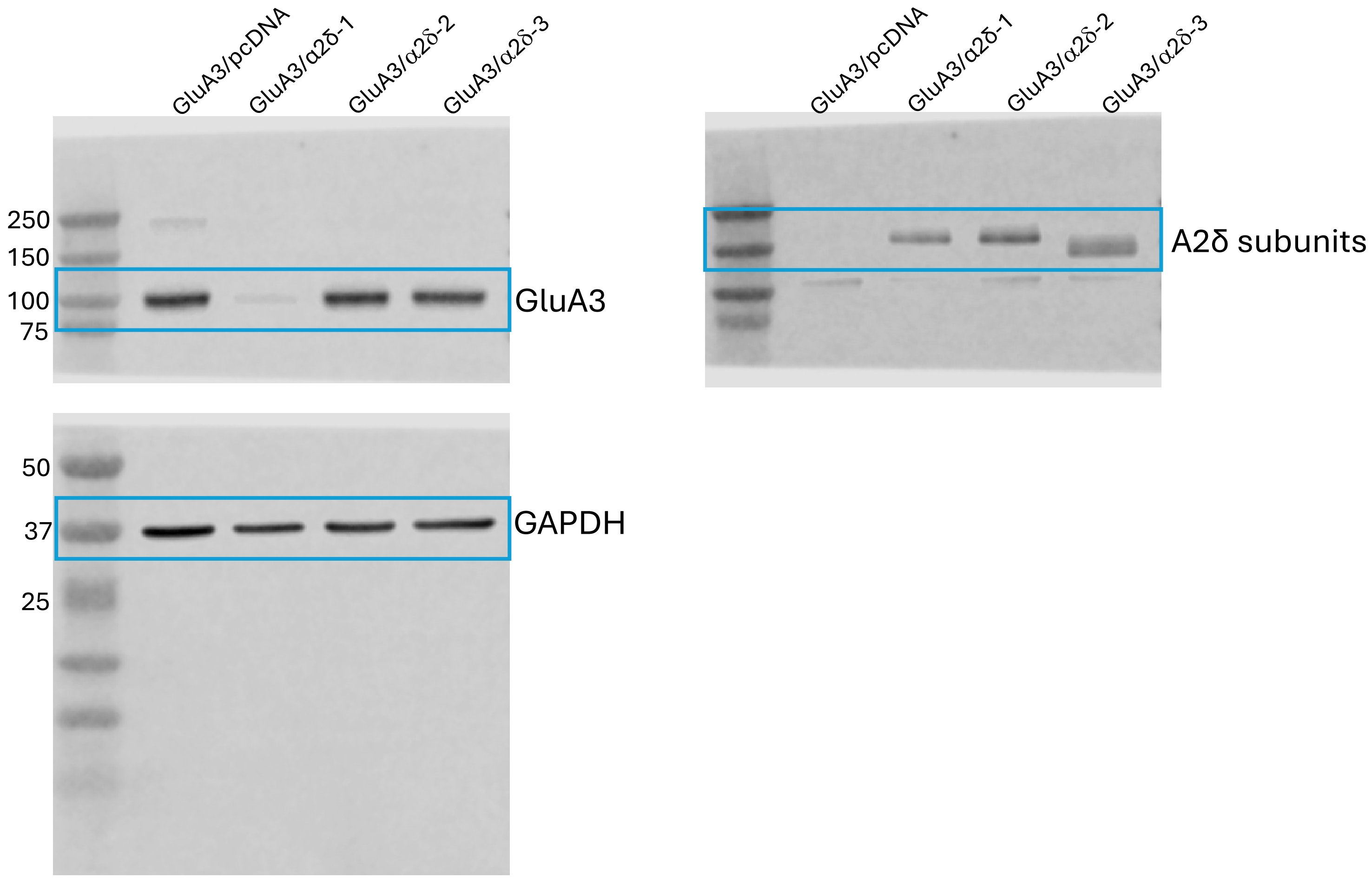

Full unedited blot for Fig. 3A

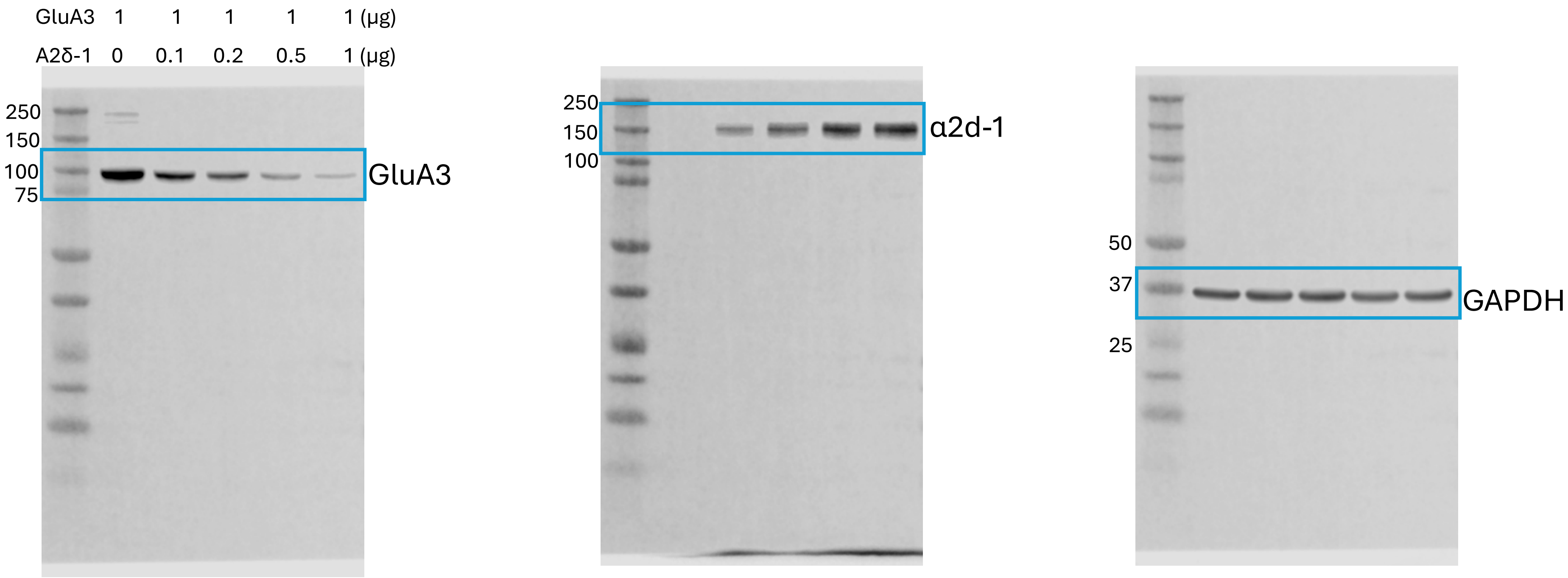

Full unedited blot for Fig. 3B

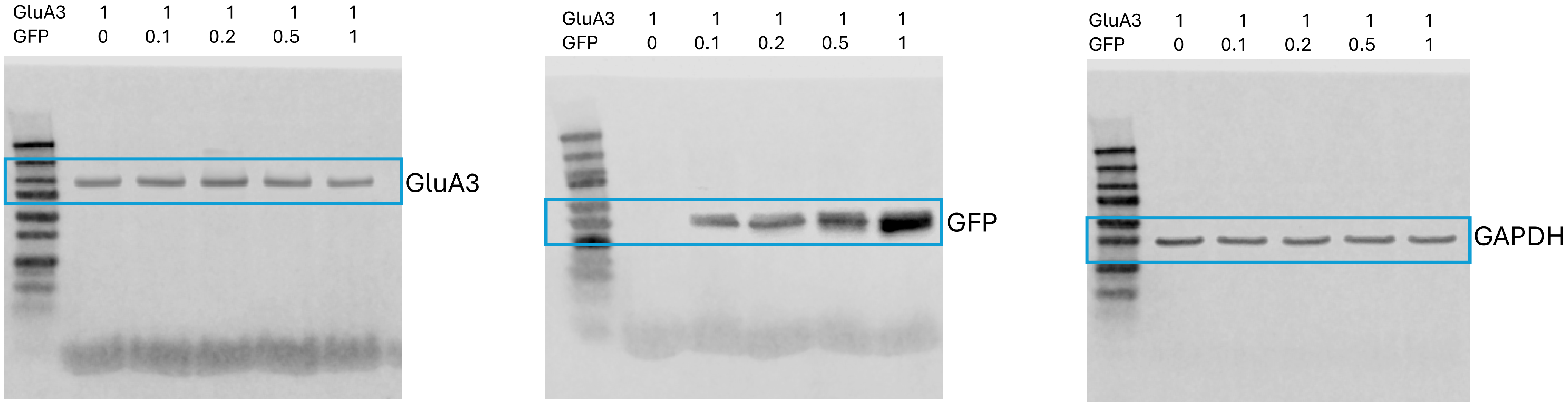

Full unedited blot for Fig. 3C

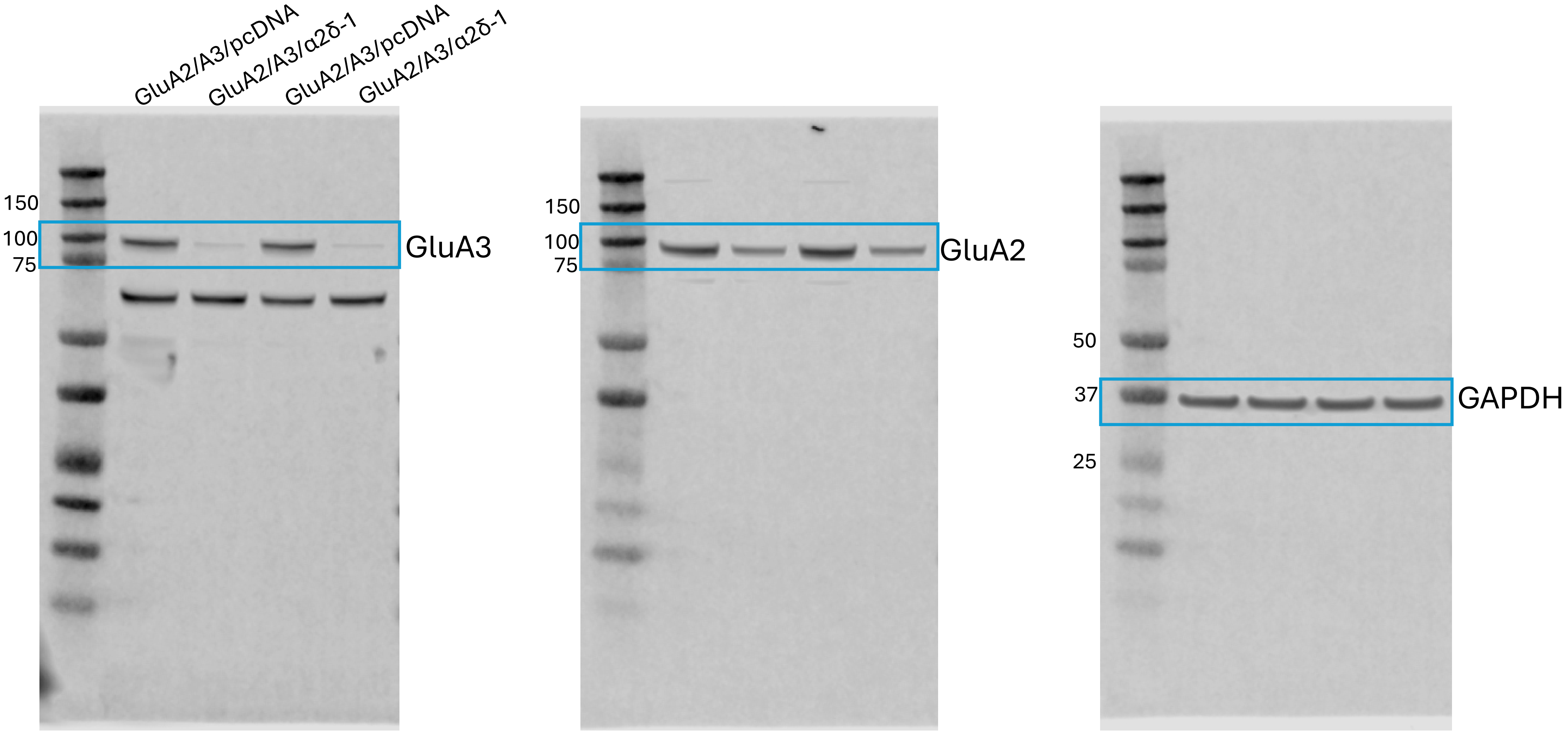

Full unedited blot for Fig. 3D

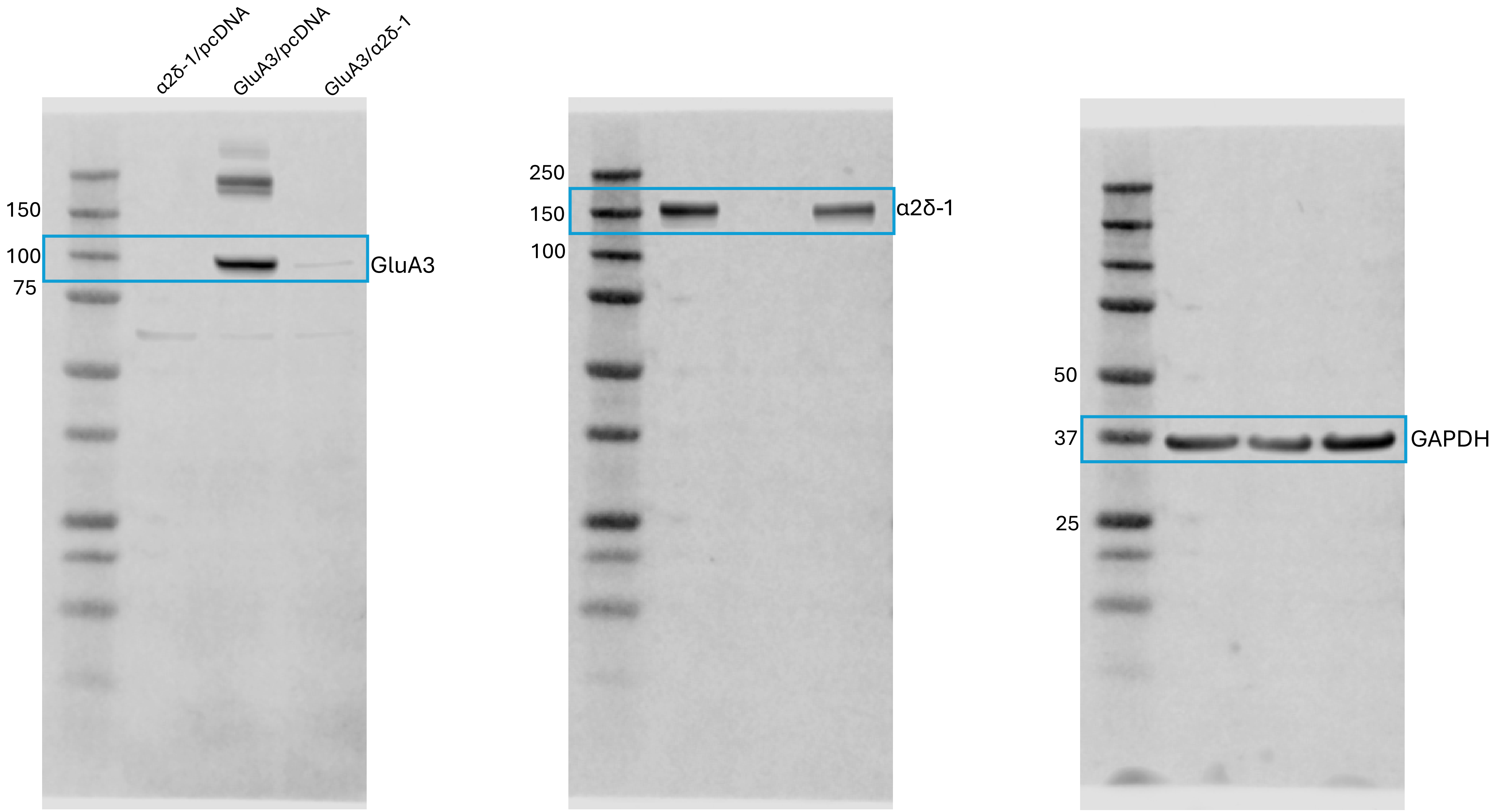

Full unedited blot for Fig. 4B

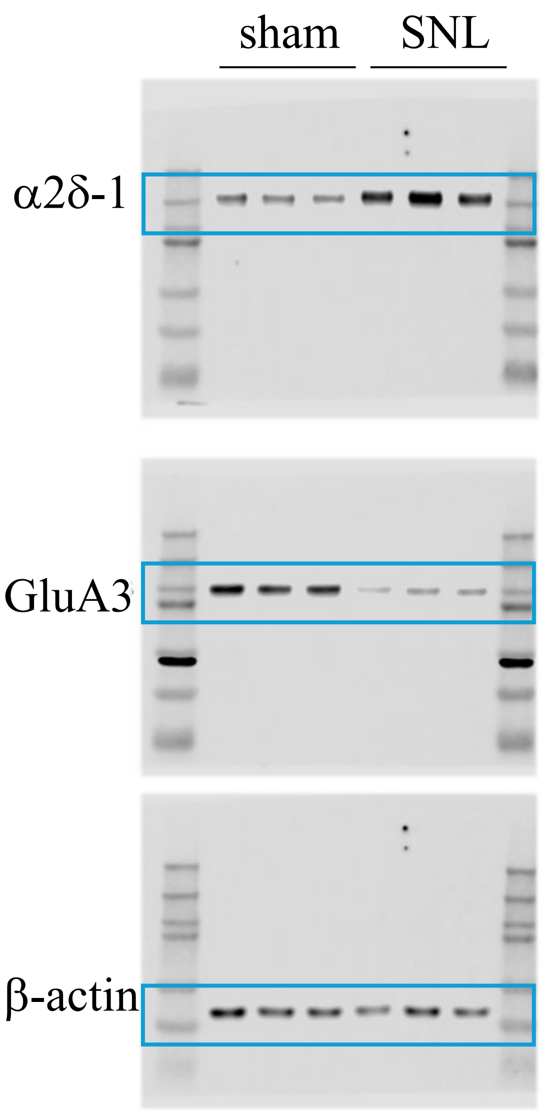

Full unedited blot for Fig. 4D

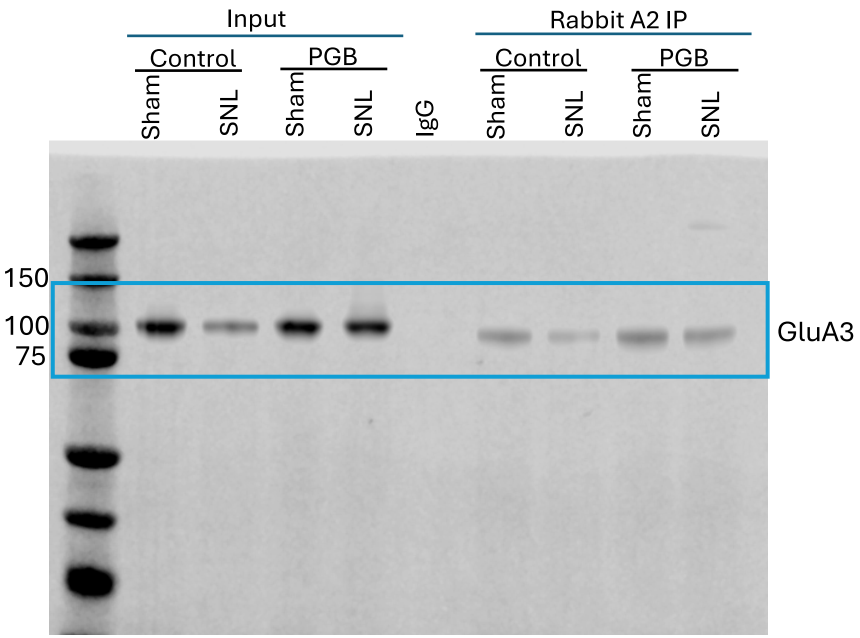

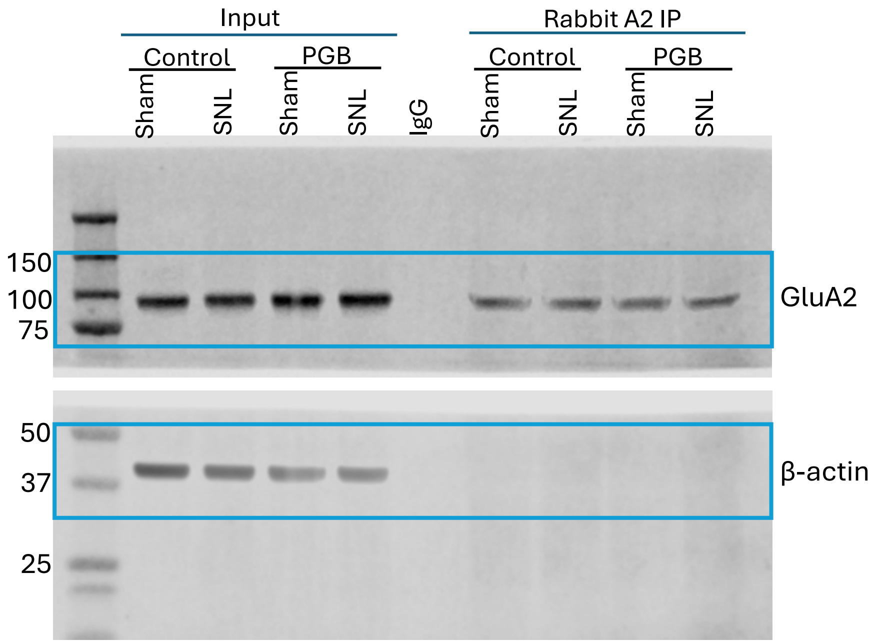

Full unedited blot for Fig. 5A

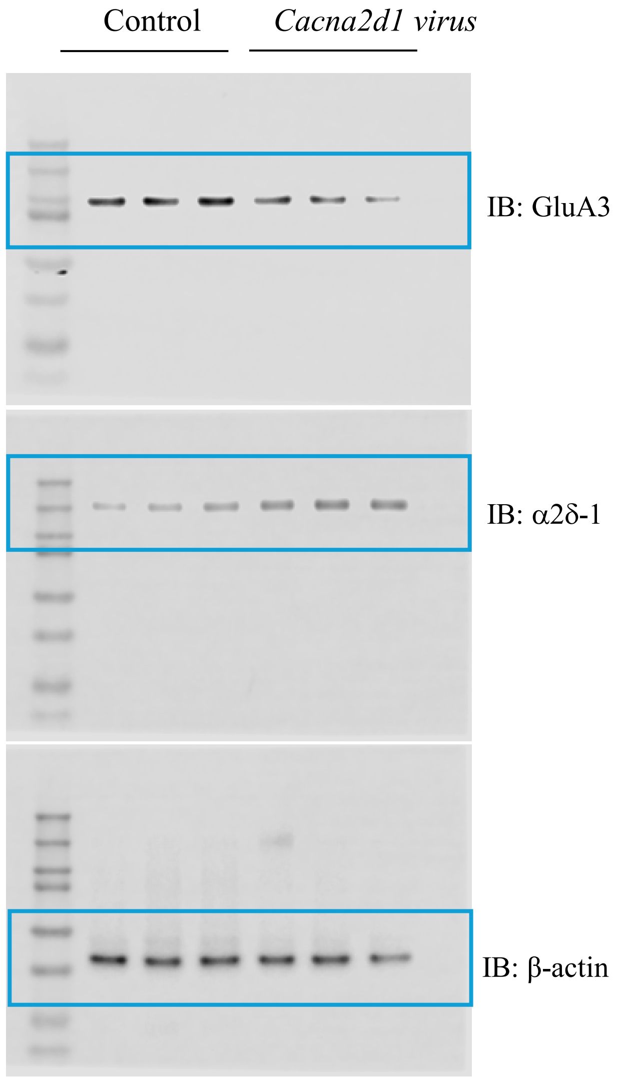

Full unedited blot for Fig. 5C

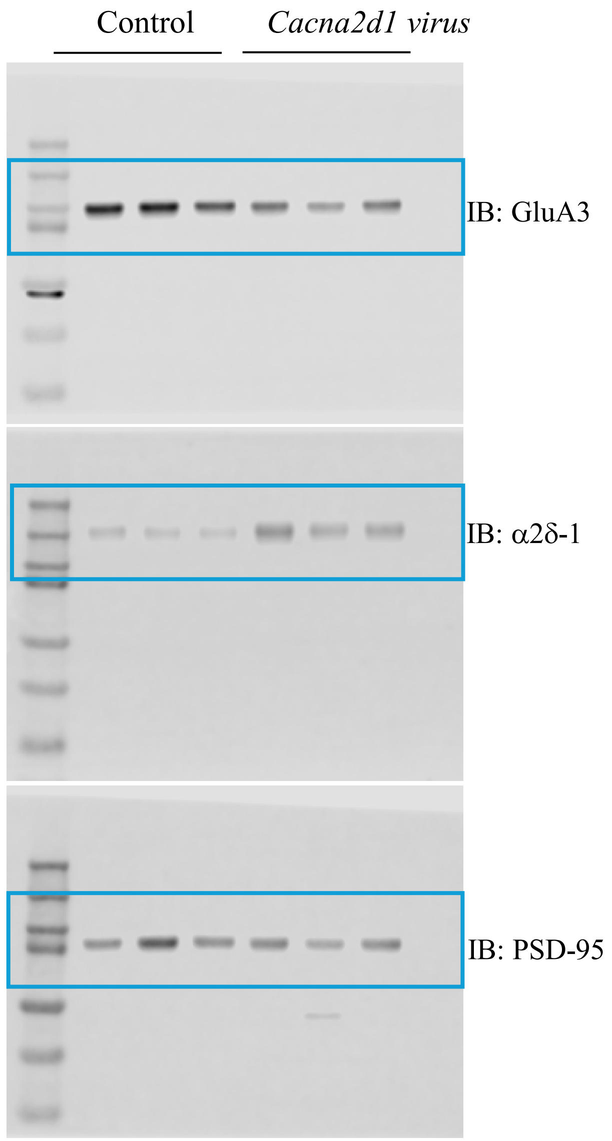

Full unedited blot for Fig. 6A

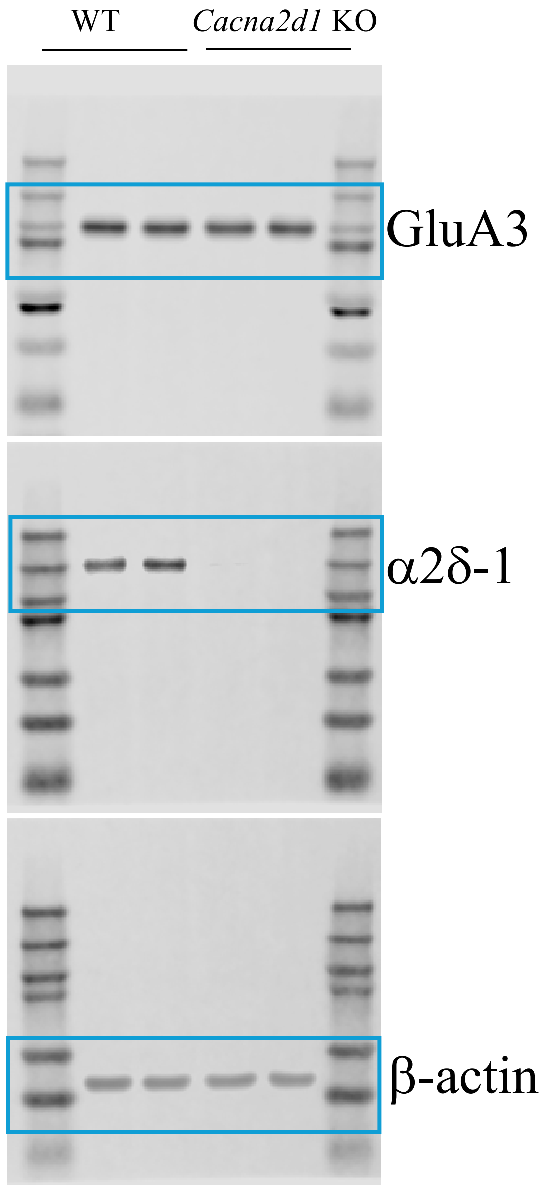

Full unedited blot for Fig. 6C

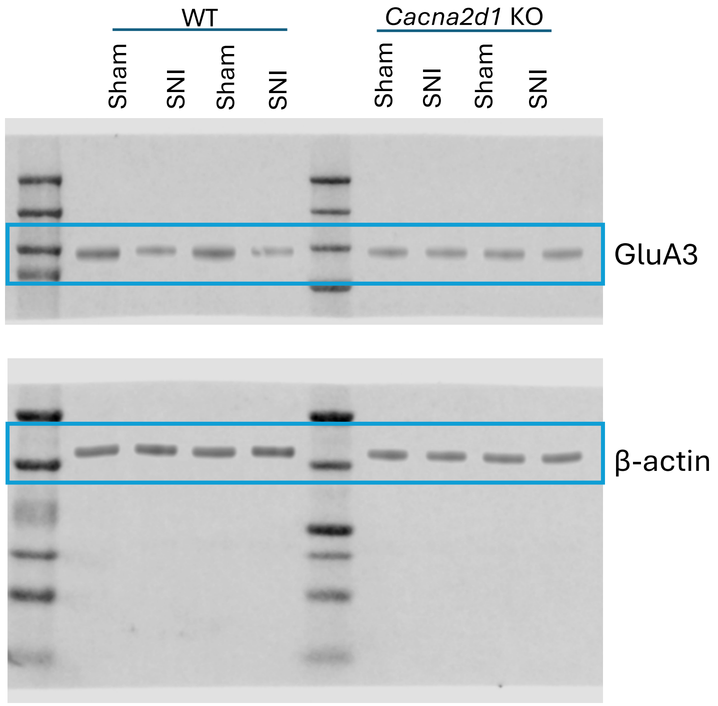

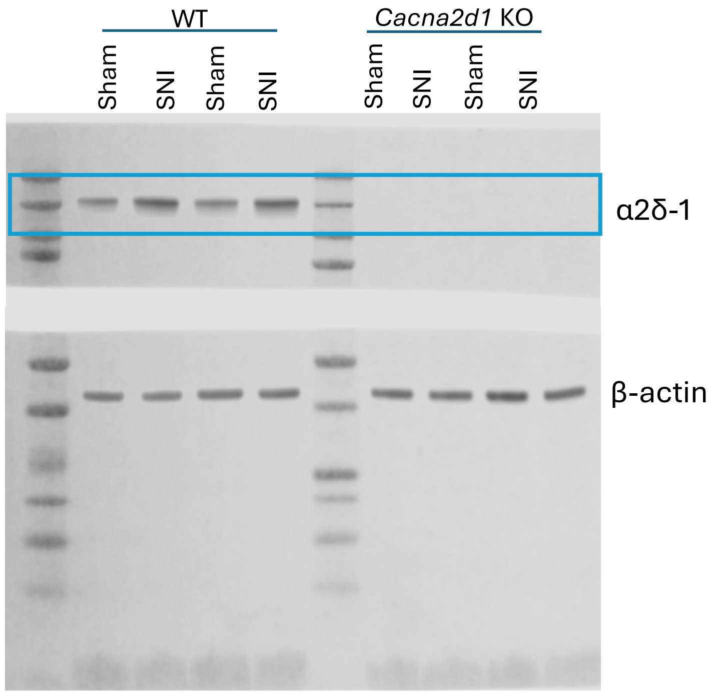

Full unedited blot for Fig. 7A

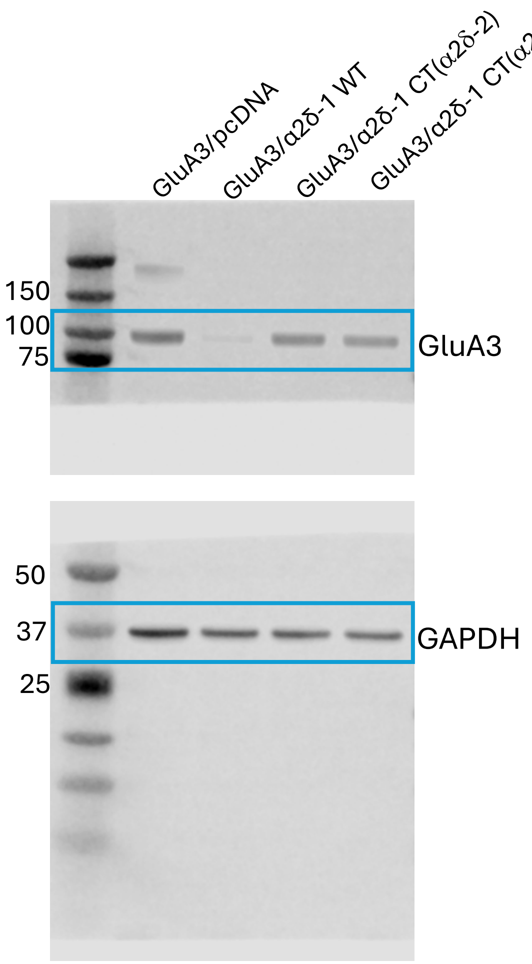

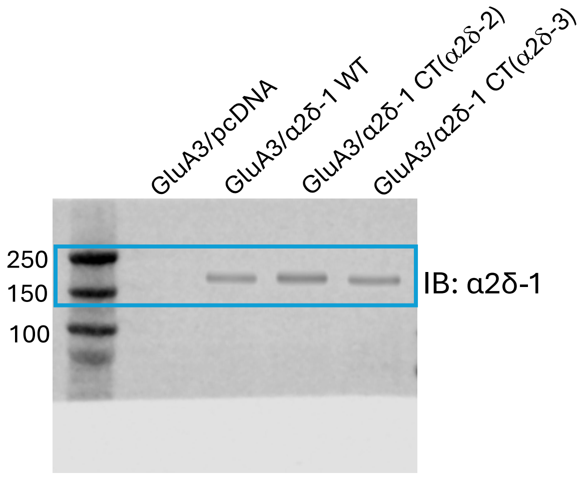

Full unedited blot for Fig. 7C

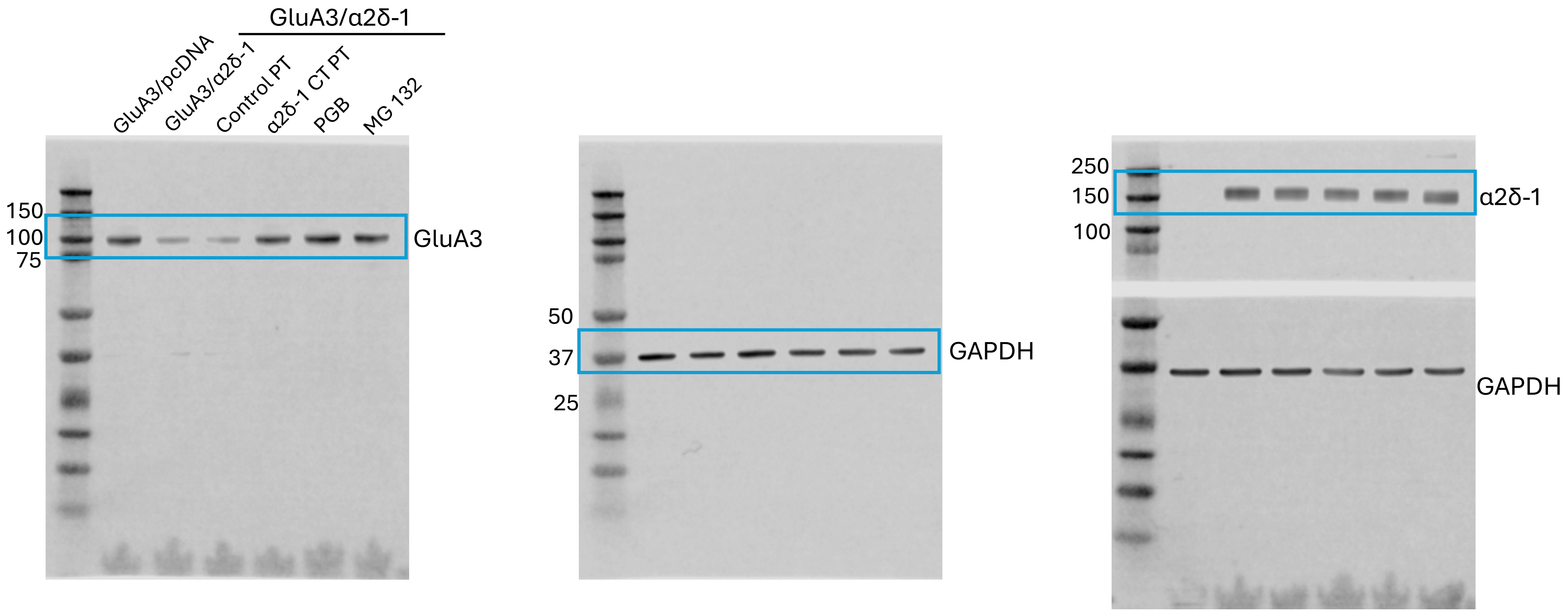

Full unedited blot for Fig. 8B

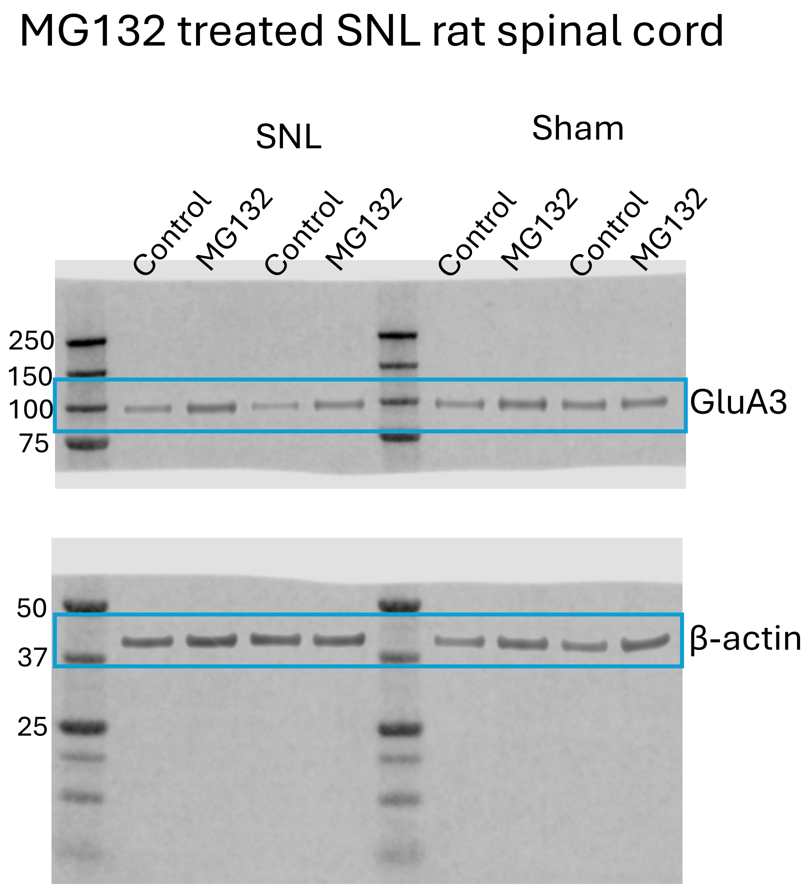

Full unedited blot for Fig. 9A

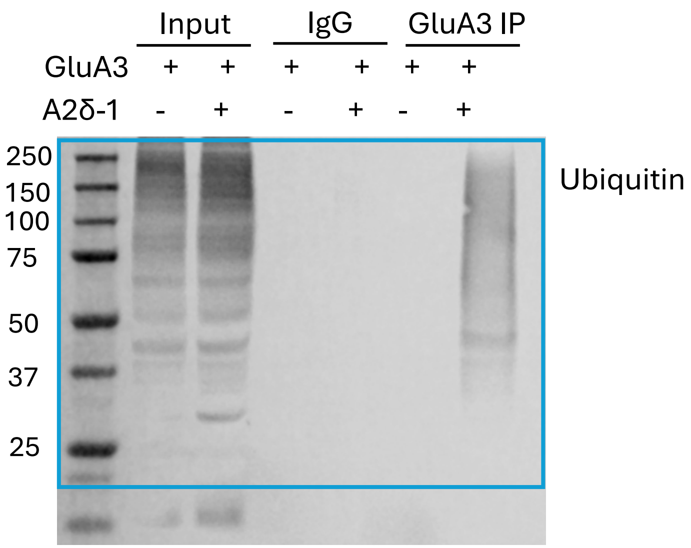

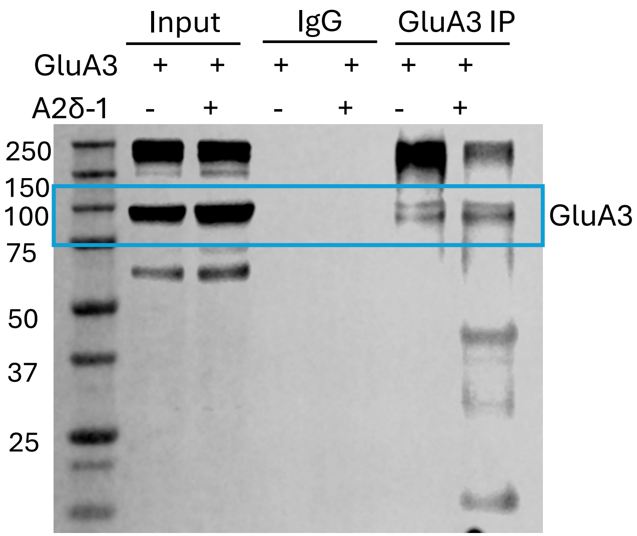

Full unedited blot for Fig. 9B

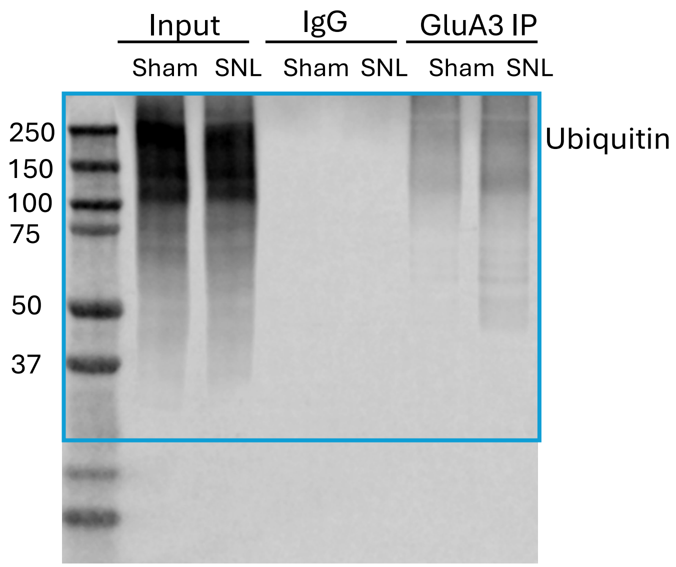

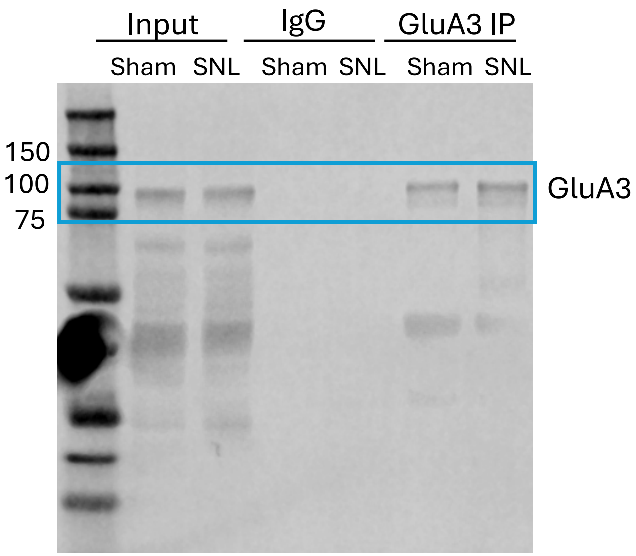

Full unedited blot for Fig. 9C

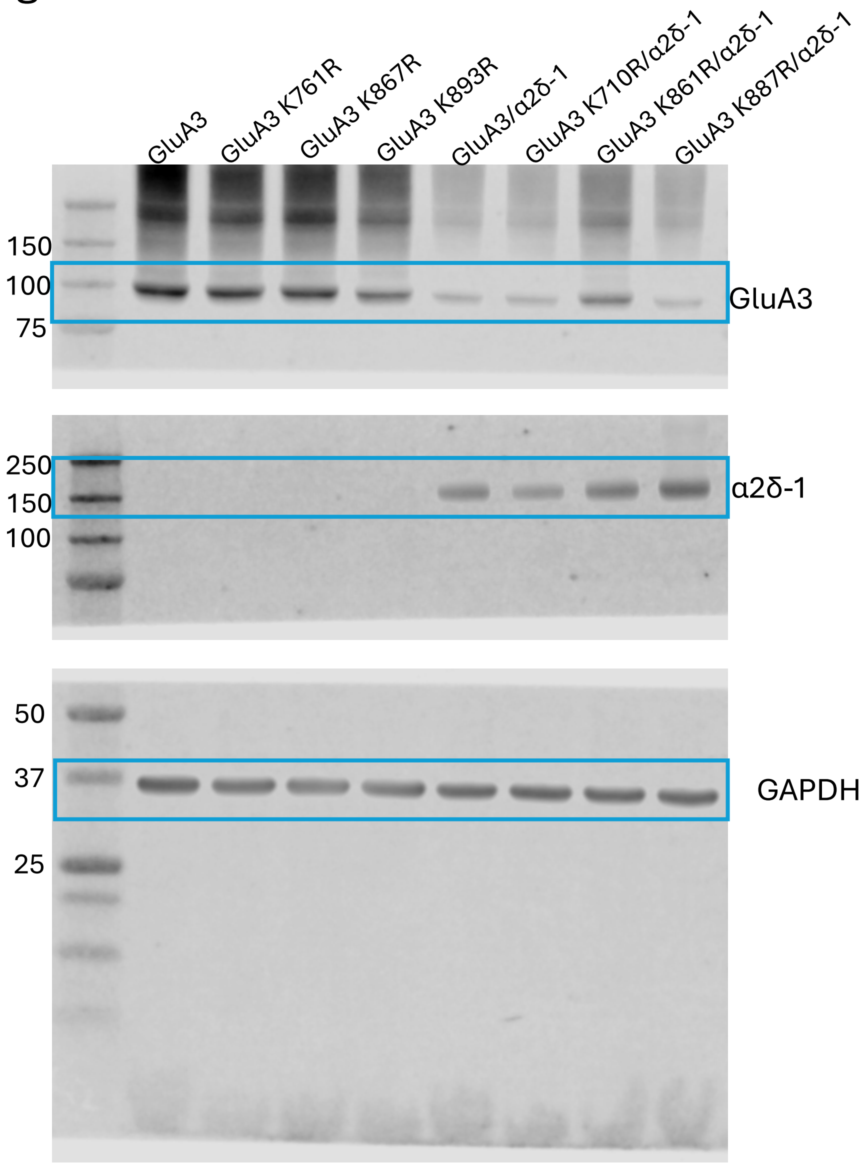

Full unedited blot for Fig. 10B

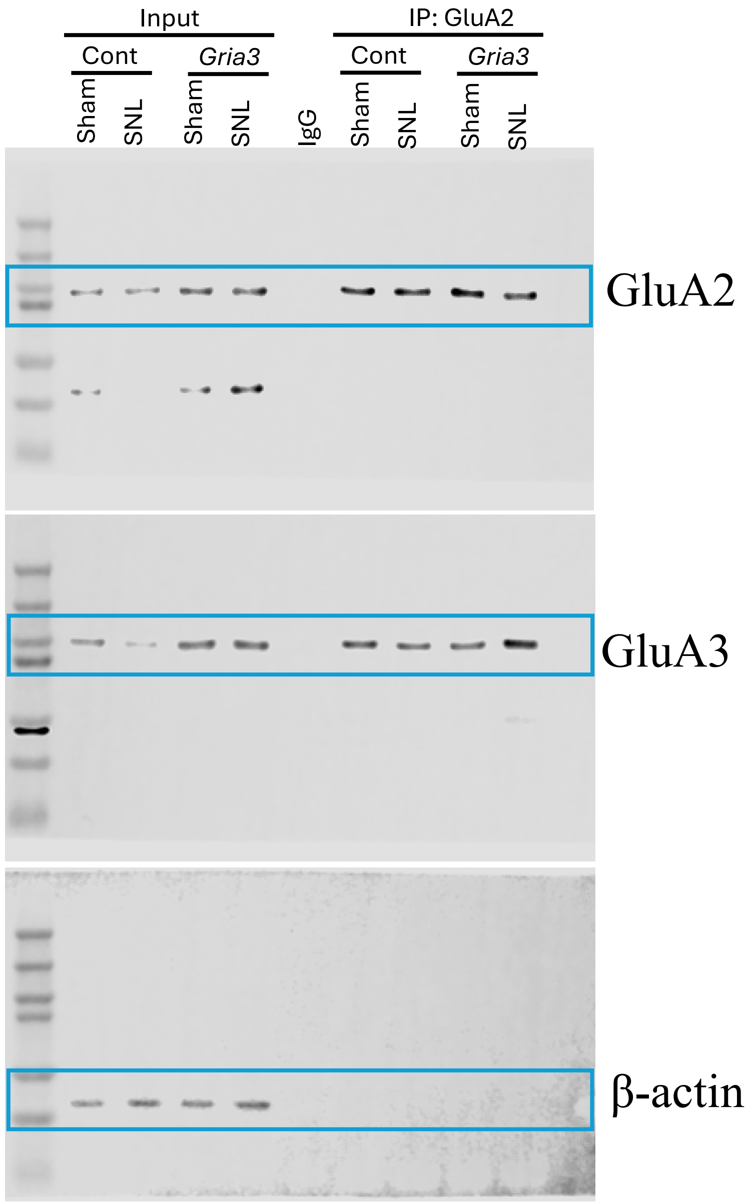

Full unedited blot for Fig. S1

α2δ-1 protein levels with GluA3 overexpression

Transfection ratio (pcDNA/α2δ-1:GluA3):

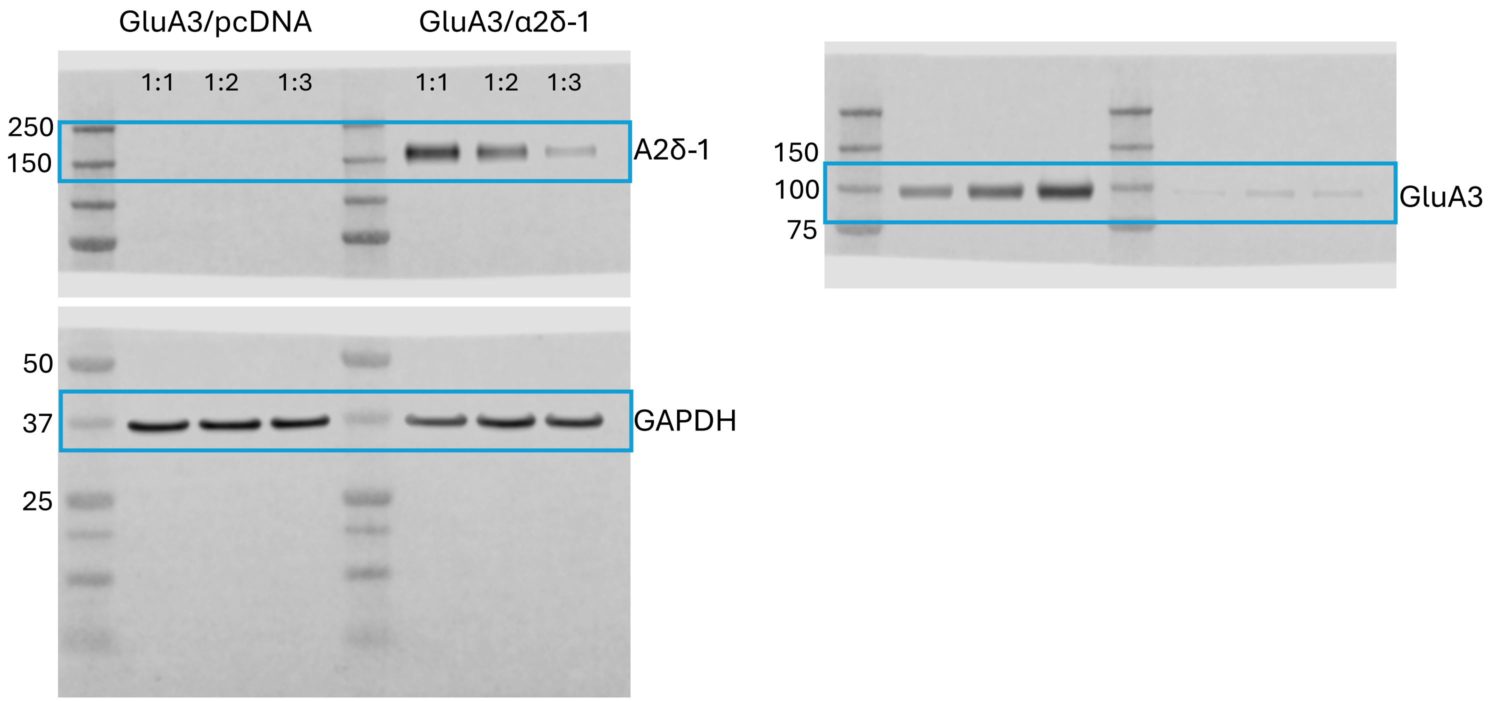

Full unedited blot for Fig. S3

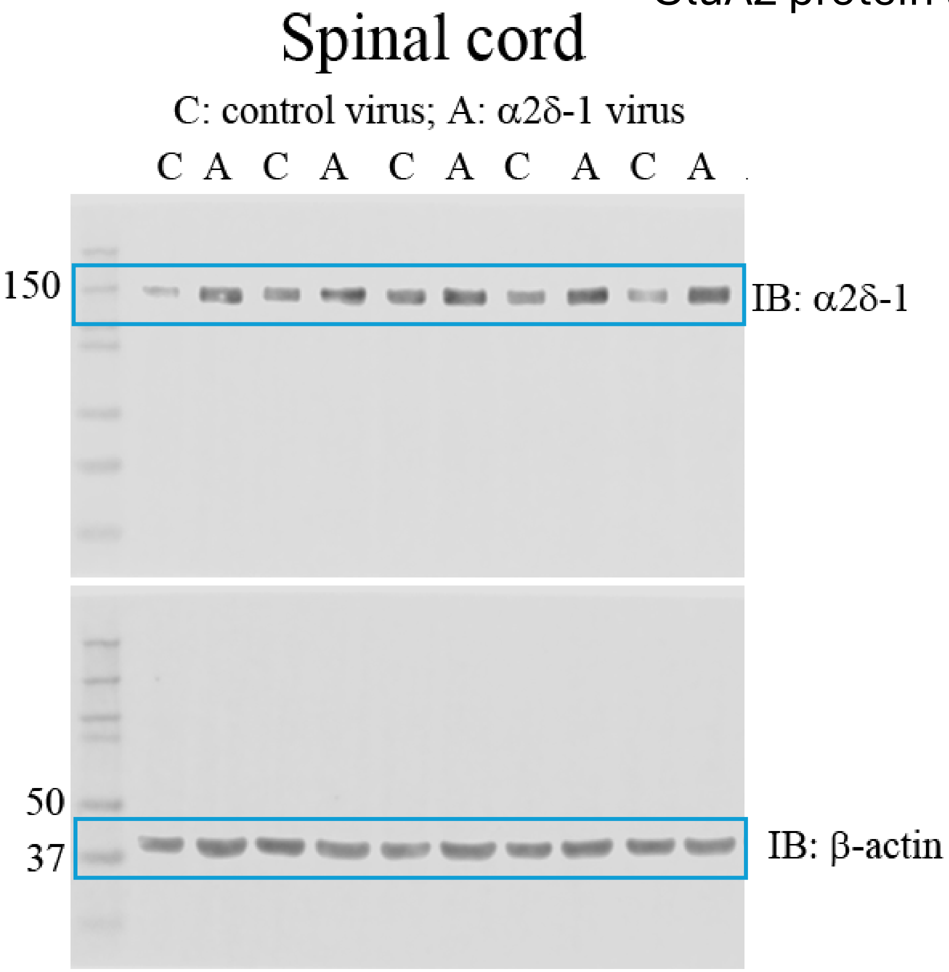

GluA2 protein levels with α2δ-1 overexpression

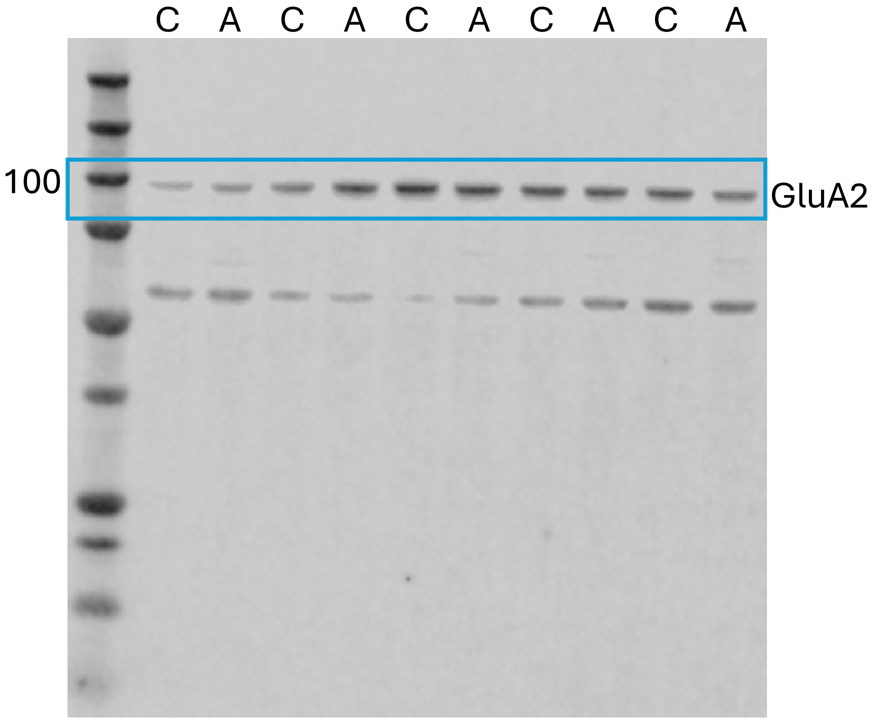

Supplement: Unedited blot and gel images [file jci-136-193349-s102.pdf]
